# Prognostic Value of Systemic Inflammation, Nutritional Status and Sarcopenia in Patients With Amyotrophic Lateral Sclerosis

**DOI:** 10.1002/jcsm.13618

**Published:** 2024-10-24

**Authors:** Yahui Zhu, Ying Zhang, Mao Li, Jiongming Bai, Hongfen Wang, Xinyuan Pang, Rongrong Du, Jiao Wang, Xusheng Huang

**Affiliations:** ^1^ Department of Neurology, The First Medical Center Chinese PLA General Hospital Beijing China; ^2^ Department of Neurology, Beijing Tiantan Hospital Capital Medical University Beijing China; ^3^ Medical School of Chinese PLA Beijing China; ^4^ Department of Health Care, The Second Medical Center Chinese PLA General Hospital Beijing China; ^5^ College of Medicine Nankai University Tianjin China

**Keywords:** amyotrophic lateral sclerosis, inflammation, nutrition, sarcopenia index, survival

## Abstract

**Background:**

Nutritional status, systemic inflammatory responses and muscle mass are associated with the prognosis of patients with amyotrophic lateral sclerosis (ALS). However, the optimal biomarker for predicting prognosis remains unclear. This study aimed to identify the optimal indicators of survival among the nutrition‐based, inflammation‐based and muscle mass–related markers for ALS patients.

**Methods:**

We enrolled ALS patients from January 2014 to December 2019. Experienced neurologists followed up with the participants until January 2022. This study included a total of 17 nutritional, systemic inflammatory or muscle mass–related indicators. Maximally selected rank statistics determined the cut‐off points for these indicators. Kaplan–Meier estimation was used to assess survival. Uni‐ and multivariate Cox proportional hazards models were used to determine the effects of indicators on survival. Finally, time‐dependent receiver operating characteristic (time‐ROC) curves and the C‐index were calculated to evaluate the predictive efficacy of different indicators.

**Results:**

A total of 506 patients with ALS were enrolled in this study, including 288 males (56.9%) and 218 females (43.1%), with a mean age of 54.2 ± 10.5 years. Among these ALS patients, 334 cases (68.0%) either died or underwent tracheotomy. In univariate Cox proportional hazards regression, 11 indicators were significantly associated with ALS survival (*p* < 0.05). And systemic immune inflammation (SII), platelet‐to‐lymphocyte ratio (PLR), modified geriatric nutritional risk index (mGNRI), creatinine and sarcopenia index (SI, (creatinine/cystatin C) × 100) were determined as independent predictors (*p* < 0.05) in multivariate Cox proportional hazards regression. A higher SI predicted longer survival (hazard ratio, 0.59; 95% confidence interval [CI], 0.46–0.76; *p* < 0.001). The results of time‐ROC and C‐index analyses indicated that SI had the best predictive efficacy for ALS survival, with a C‐index of 0.65 (95% CI, 0.54–0.75) for 1‐year, 0.61 (95% CI, 0.57–0.65) for 3‐year and 0.59 (95% CI, 0.55–0.62) for 5‐year survival. Across different subgroups, SI had the highest C‐index in men and women, limb onset and aged < 60 year ALS patients, compared with other indicators. However, cystatin C was the best indicator for predicting the survival of ALS patients with bulbar onset, whereas the prognostic nutritional index (PNI) was the best for those aged ≥60 years.

**Conclusions:**

The serum SI demonstrates superior prognostic ability compared to other inflammation‐based, nutrition‐based and muscle mass–related indicators for patients with ALS. Given its simplicity and availability, it is well suited for clinical use in evaluating the prognosis of ALS patients.

## 1 Introduction

Amyotrophic lateral sclerosis (ALS) is a neurodegenerative disease characterized by progressive muscle weakness and atrophy due to the loss of motor neurons in the brain, brainstem and spinal cord [1]. ALS can be categorized as bulbar onset or spinal onset based on the initial site of symptoms. Patients with spinal onset initially present with weakness in one region of the body, which gradually spreads to other areas over time. Most ALS patients succumb within 2–4 years of onset, although a small subset may experience a slower‐progressive form of the disease [2].

Not only the diagnosis of ALS is challenging, but also the prognosis of patients with ALS remains suboptimal due to the incomplete understanding of the disease progression. Efforts to improve early diagnosis and prognosis in ALS include investigating new ALS criteria and scoring systems, as well as emerging biofluid markers, imaging techniques and electrophysiological measures [2]. Neurofilaments, neuronal cytoskeleton proteins involved in maintaining neuronal shape, have been identified as potential biomarkers. Specifically, phosphorylated neurofilament heavy chain (pNFH) in cerebrospinal fluid (CSF) and phosphorylated neurofilament light chain (NFL) in plasma, serum or CSF have been found to be elevated in ALS patients compared to the control group [3]. Higher levels of pNFH and NFL in ALS patients are associated with more aggressive disease progression and shorter survival, although their prognostic value is limited [3, 4]. Furthermore, elevated neurofilament levels are frequently observed in other neurodegenerative diseases [5]. Therefore, although neurofilaments alone may not be enough to diagnose or predict the prognosis, they may offer enhanced value when combined with other approaches.

Advanced central nervous system imaging techniques provide additional prognostic insights that can complement current methods [6, 7, 8]. However, further studies are needed to evaluate their integration into clinical practice. In terms of electrophysiological measurements, hyperexcitability by transcranial magnetic stimulation has shown some prognostic utility [9, 10, 11].

Recently, growing evidence suggested that various inflammation‐ and nutrition‐related factors can serve as effective prognostic predictors. Markers of systemic inflammatory response, including plasma C‐reactive protein (CRP) levels [12] and neutrophil‐to‐lymphocyte ratio (NLR) [13], have been demonstrated to be crucial in the progression and prognosis of ALS patients. Systemic immune inflammation (SII) has also been found to be associated with a more rapid disease progression [14]. Moreover, nutrition‐based indicators, such as the geriatric nutritional risk index (GNRI), have been found negatively associated with disease severity [15]. However, the prognostic significance of other inflammation/nutrition markers, such as the advanced lung cancer inflammation index (ALI), the CRP to albumin ratio (CAR), the inflammatory burden index (IBI), the prognostic nutritional index (PNI) and the nutritional risk index (NRI), remains unknown.

Muscle atrophy is a hallmark of ALS. Although it serves as a qualitative indicator of disease progression, a reliable quantitative marker of atrophy has yet to be identified. Therefore, identifying a parameter that reflects muscle mass and aids in estimating disease severity and prognosis is imperative. Serum creatinine might reflect muscle wasting and serve as an independent marker of ALS outcome in both sexes [16]. However, as serum creatinine is predominantly derived from skeletal muscle and is influenced by renal function, its accuracy as a marker for ALS can be questioned. Cystatin C, a cysteine protease inhibitor produced by all nucleated cells, serves as a surrogate marker of glomerular filtration rate (GFR) [17, 18]. It is considered to be unaffected by factors, such as muscle mass, lean tissue mass, age, activity, circadian rhythm and sex, except for renal function status [19, 20]. Additionally, cystatin C is independent of body muscle mass and is excreted by the kidneys like creatinine. In our previous study, we proposed that serum cystatin C is associated with ALS survival and may serve as an independent prognostic predictor [21]. Kashani et al. utilized the differential cellular origins of creatinine and cystatin C to develop a new index, sarcopenia index (SI, (serum creatinine/serum cystatin C) × 100) to assess muscle mass, using the surface area of paraspinal muscles at the level of the fourth lumbar vertebrae on CT scans as the gold standard [22]. They found a significant correlation between the SI and measured muscle mass via CT scan [22]. A low SI suggests a reduction in the skeletal muscle necessary to produce creatinine when the effects of renal function are corrected, thus indicating sarcopenia. However, the prognostic significance of SI in ALS remains unknown.

Therefore, this study aimed to assess the value of various inflammation‐, nutrition‐ and muscle mass–related indicators for predicting the survival of ALS patients and to identify the optimal prognostic indicator for ALS patients.

## 2 Methods

### 2.1 Participants

In this study, all the patients included met the revised El Escorial Criteria of ALS [23]. The outpatients under consideration for an ALS diagnosis were admitted to the ward. The patients were recruited upon finishing the relevant tests and meeting the diagnostic criteria [23]: (A) the presence of (A:1) evidence of lower motor neuron (LMN) degeneration by clinical, electrophysiological or neuropathologic examination, (A:2) evidence of upper motor neuron (UMN) degeneration by clinical examination, and (A:3) progressive spread of symptoms or signs within a region or to other regions, as determined by history or examination, together with (B) the absence of (B:1) electrophysiological or pathological evidence of other disease processes that might explain the signs of LMN and/or UMN degeneration and (B:2) neuroimaging evidence of other disease processes that might explain the observed clinical and electrophysiological signs. The clinical diagnostic certainty on ALS clinical criteria is divided into four categories depending on the presence of upper motor neuron (UMN) and lower motor neuron (LMN) signs together in the same topographical anatomic region in either the brainstem (bulbar cranial motor neurons) or the cervical, thoracic or lumbosacral spinal cord (anterior horn motor neurons): clinically definite, clinically probable, clinically probable laboratory supported and clinically possible ALS. In this study, we investigated sporadic ALS patients (including clinically definite, clinically probable, clinically probable laboratory supported and clinically possible ALS patients) diagnosed at the Department of Neurology, The First Medical Center, Chinese PLA General Hospital, from January 2014 to December 2019 based on the revised El Escorial Criteria [23].

ALS patients with evidence of systemic diseases (malignant tumour, rheumatoid arthritis and hepatic or renal dysfunction), as well as those who recently (≤ 3 months) received glucocorticoid therapy or had acute cardiovascular events, were excluded. In addition, patients with missing data of inflammation, nutrition, and muscle mass indicators were excluded.

This study was conducted in compliance with the Declaration of Helsinki and approved by the Ethics Committee of Chinese PLA General Hospital. Written informed consent was obtained from participants.

### 2.2 Baseline Clinical Data

Baseline clinical data of ALS patients, including gender, age, sites of onset, diagnostic delay, body mass index (BMI) and ALSFRS‐R score, were obtained. Sites of onset was categorized as bulbar onset and limb onset. Diagnostic delay was defined as the time interval (in months) between symptom onset and diagnosis. BMI was calculated as weight in kilograms divided by height in meters squared. ALS patients were evaluated by experienced neurologists within 24 h after admission with the ALSFRS‐R scale.

### 2.3 Measurements of Inflammation/Nutrition‐Based and Muscle Mass–Related Indicators in Routine Blood Tests

Routine blood tests were obtained after fasting for at least 9 h, within 24 h of hospitalization, and included the levels of glucose, albumin, globulin, CRP, creatinine, cystatin C and lymphocyte, neutrophil and platelet counts. The inflammation/nutrition‐based indicators and muscle mass–related indicators used in this study included the ALI, SII, platelet‐to‐lymphocyte ratio (PLR), NLR, CRP, CAR, lymphocyte‐to‐CRP ratio (LCR), albumin‐to‐globulin ratio (AGR), IBI, PNI, glucose‐to‐lymphocyte ratio (GLR), GNRI, modified GNRI (mGNRI), NRI, creatinine, cystatin C and SI. The calculation methods for the combination of each inflammation/nutrition‐based and muscle mass–related indicator were shown in Table S1.

### 2.4 Follow‐Up Study

The participants were regularly followed by experienced neurologists with telephone or face‐to‐face interview every 6 months until January 2022. The survival time of patients was defined as disease onset to death, tracheostomy or censoring date (31 January 2022). Furthermore, for patients who were lost to follow‐up, survival time was calculated as onset to last contact. If the neurologists failed to contact participants twice by phone, patients were considered to be lost to follow‐up.

### 2.5 Statistical Analysis

For comparisons of continuous variables, unpaired *t*‐test was used if both samples had passed the Shapiro–Wilk test for normality; otherwise, the Mann–Whitney *U* test was used. We used χ^2^ test to compare frequencies for categorical variables. We dichotomized the continuous inflammation/nutrition‐based and muscle mass–related indicators based on the optimal cut‐off points calculated using maximally selected rank statistics. Survival comparison between groups were performed with Kaplan–Meier curves and log‐rank tests. Uni‐ and multivariate Cox proportional hazards regression analyses were conducted to identify variables related to ALS survival. The predictive accuracy of each indicator was assessed using the C‐index and time‐dependent receiver operating characteristic (time‐ROC) curves. In addition, to determine whether the same indicators were applicable to all subgroups and to gain insight into the most useful biomarkers in different subgroups, we performed subgroup analyses by age, sex and site of onset in ALS patients. Data for continuous variables were summarized and recorded as mean ± standard deviation (SD) or median with quartile. All tests were two‐tailed with a statistical significance level of *p* < 0.05. All statistical analyses were carried out using SPSS Version 22 software and R Version 4.1.2, including the R packages ‘survival’, ‘survminer’, ‘rms’, ‘timeROC’, ‘maxstat’, ‘ggplot2’ and ‘forestplot’.

## 3 Results

### 3.1 Participant Characteristics

**FIGURE 1:**
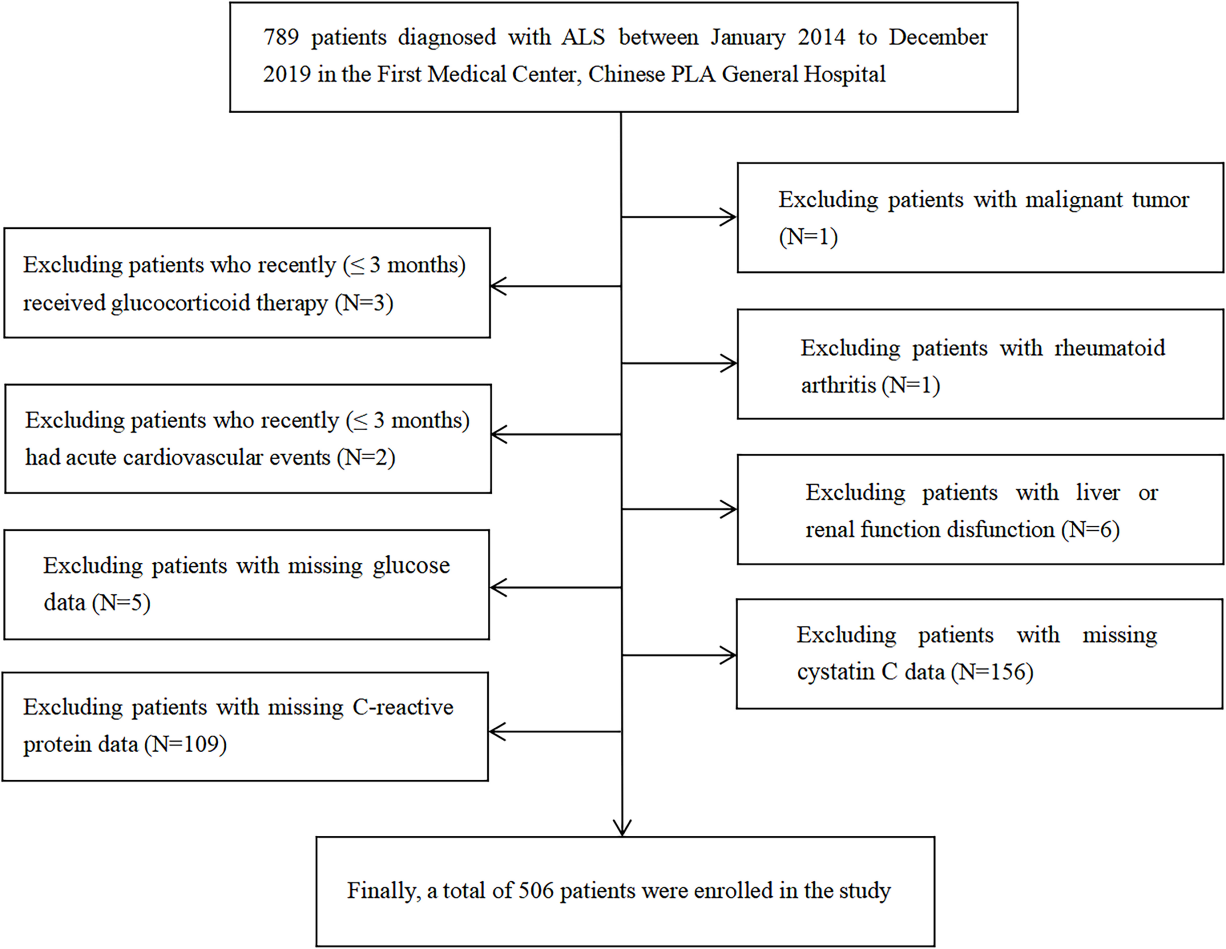
The flow chart of the participants selection.

According to the above inclusion and exclusion criteria, we initially recruited 789 patients with ALS from January 2014 to December 2019 and then excluded 283 patients. Therefore, 506 patients with sporadic ALS were enrolled, including 288 males and 218 females. The flow chart of the participant selection was shown in Figure 1. The numbers of clinically definite, clinically probable, clinically probable laboratory supported and clinically possible ALS patients were 171 (33.8%), 198 (39.1%), 114 (22.5%) and 23 (4.5%), respectively. There were 88 (17.4%) cases of bulbar onset and 418 (82.6%) patients of limb onset. The mean age was 54.2 years old, and the mean BMI was 23.4. The median time interval between symptom onset and diagnosis was 11.0 months. The median ALSFRS‐R score was 40 (Table 1).

**TABLE 1:** Clinical features of ALS patients.

	All patients
Number	506
Sex (men/women)	288/218
Site of onset (bulbar/limb)	88/418
Age (years)	54.2 ± 10.5
Diagnostic delay (months)	11.0 (6.0–18.0)
BMI	23.4 ± 3.1
ALSFRS‐R score	40 (36–44)
Total protein (g/L)	64.9 (61.9–68.0)
Albumin (g/L)	41.4 (39.3–43.3)
Glucose (mmol/L)	4.70 (4.37–5.11)
Neutrophil (×10^9^/L)	3.29 (2.67–4.06)
Lymphocyte (×10^9^/L)	1.87 (1.54–2.28)
Platelets (×10^9^/L)	216 (184–257)
C‐reactive protein (mg/L)	1.00 (1.00–3.23)
Creatinine (umol/L)	60.4 (51.8–69.4)
Cystatin C (mg/L)	0.98 (0.86–1.10)
ALI	55.89 (41.32–71.17)
SII	374.79 (281.38–518.09)
PLR	117.84 (94.76–146.10)
NLR	1.74 (1.34–2.24)
CAR	0.026 (0.023–0.079)
LCR	13153.25 (6181.49–21985.30)
IBI	0.27 (0.15–0.56)
AGR	1.76 (1.60–1.93)
PNI	50.75 (48.05–53.51)
GLR	2.58 (2.05–3.19)
GNRI	102.45 (98.88–105.43)
mGNRI	54.88 (46.13–56.59)
NRI	103.68 (100.03–106.71)
SI	69.2 (59.8–79.8)
Riluzole (yes/no)	230/276
Edaravone (yes/no)	344/162

*Note:* Data for continuous variables were summarized and recorded as mean ± standard deviation (SD) or median with interquartile range (IQR). The age and BMI were recorded as mean ± SD, and other continuous variables were summarized as median with IQR.

Abbreviations: AGR, albumin‐to‐globulin ratio; ALI, advanced lung cancer inflammation index; ALS, amyotrophic lateral sclerosis; BMI, body mass index; CAR, C‐reactive protein‐to‐albumin ratio; GLR, glucose‐to‐lymphocyte ratio; GNRI, geriatric nutritional risk index; IBI, inflammatory burden index; LCR, lymphocyte‐to‐C‐reactive protein ratio; mGNRI, modified geriatric nutritional risk index; NLR, neutrophil‐to‐lymphocyte ratio; NRI, nutritional risk index; PLR, platelet‐to‐lymphocyte ratio; PNI, prognostic nutritional index; SI, sarcopenia index; SII, systemic immune inflammation index.

### 3.2 Association of Inflammation/Nutrition‐Based Indicators and Muscle Mass–Related Indicators and Survival in ALS Patients

**FIGURE 2:**
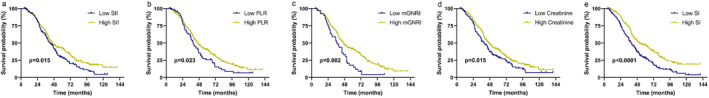
The Kaplan–Meier curves in patients with amyotrophic lateral sclerosis of SII (a), PLR (b), mGNRI (c), creatinine (d), and SI (e). mGNRI, modified geriatric nutritional risk index; PLR, platelet‐to‐lymphocyte ratio; SI, sarcopenia index; SII, systemic immune inflammation index.

After nearly 8 years of follow‐up, 344 cases (68.0%) either died or underwent tracheostomy, and the median survival time was 45.0 months. The cut‐off points of inflammation/nutrition‐based and muscle mass–related indicators were 56.51 (ALI), 317.85 (SII), 84.95 (PLR), 1.18 (NLR), 0.74 (CRP), 0.023 (CAR), 4121.04 (LCR), 0.27 (IBI), 1.51 (AGR), 45.91 (PNI), 1.96 (GLR), 98.71 (GNRI), 43.36 (mGNRI), 99.76 (NRI), 53.0 (creatinine), 1.05 (cystatin C) and 68.8 (SI). Uni‐ and multivariate analyses showed that the SII, PLR, mGNRI, creatinine and SI, but not ALI, NLR, CRP, CAR, LCR, IBI, AGR, PNI, GLR, GNRI, NRI and cystatin C, were independent predictive factors for ALS survival (Table 2). Figure 2 showed the Kaplan–Meier curves of the SII, PLR, mGNRI, creatinine and SI in all ALS patients. The median survival time of low SI and high SI of ALS patients was 40.0 and 52.0 months, respectively.

**TABLE 2:** Univariate and Multivariate Cox survival analysis in all ALS patients.

		Model 0		Model 1		Model 2	
Variables	Number of patients	Hazard ratio (95% CI)	*p*	Hazard ratio (95% CI)	*p*	Hazard ratio (95% CI)	*p*
ALI							
< 56.51	257	1	0.048	/	0.497	/	0.579
≥ 56.51	249	0.81 (0.65–1.00)		/		/	
SII							
< 317.85	178	1	0.016	1	0.014	1	0.016
≥ 317.85	328	0.77 (0.62–0.95)		0.76 (0.61–0.95)		0.76 (0.61–0.95)	
PLR							
< 84.95	87	1	0.025	1	0.004	1	< 0.001
≥ 84.95	419	0.73 (0.56–0.96)		0.67 (0.51–0.88)		0.61 (0.46–0.80)	
NLR							
< 1.18	73	1	0.066	1	0.032	/	0.143
≥ 1.18	433	0.77 (0.58–1.02)		0.73 (0.55–0.97)		/	
CRP							
< 0.74	110	1	0.117	/	0.115	/	0.532
≥ 0.74	396	1.36 (0.93–1.98)		/		/	
CAR							
< 0.023	142	1	0.339	/	0.444	/	0.802
≥ 0.023	364	1.16 (0.85–1.58)		/		/	
LCR							
< 4121.04	91	1	0.273	/	0.939	/	0.780
≥ 4121.04	415	0.82 (0.58–1.17)		/		/	
IBI							
< 0.75	405	1	0.338		0.320		0.128
≥ 0.75	101	0.85 (0.61–1.19)					
AGR							
< 1.51	82	1	0.002	/	0.237	/	0.135
≥ 1.51	424	0.66 (0.51–0.86)		/		/	
PNI							
< 45.91	51	1	< 0.001	/	0.102	/	0.080
≥ 45.91	455	0.53 (0.38–0.73)		/		/	
GLR							
< 1.96	99	1	0.248	/	0.835	/	0.798
≥ 1.96	407	1.17 (0.89–1.54)		/		/	
GNRI							
< 98.71	118	1	0.003	/	0.468	/	0.494
≥ 98.71	388	0.69 (0.54–0.89)		/		/	
mGNRI							
< 43.36	88	1	0.002	/	0.398	1	0.030
≥ 43.36	418	0.57 (0.40–0.81)		/		0.68 (0.47–0.96)	
NRI							
< 99.76	116	1	0.007	/	0.618	/	0.649
≥ 99.76	390	0.71 (0.56–0.91)		/		/	
Creatinine							
< 53.0	145	1	0.017	1	0.003	1	< 0.001
≥ 53.0	361	0.76 (0.60–0.95)		0.69 (0.54–0.88)		0.60 (0.47–0.78)	
Cystatin C							
< 1.05	320	1	< 0.001	1	0.006	/	0.104
≥ 1.05	186	1.61 (1.30–1.99)		1.37 (1.10–1.72)		/	
SI							
< 68.8	247	1	< 0.001	1	0.002	1	< 0.001
≥ 68.8	259	0.60 (0.48–0.74)		0.69 (0.54–0.88)		0.59 (0.46–0.76)	

*Note:* Model 0, unadjusted model; Model 1, adjusted by age, sex and BMI (except for ALI); Model 2, adjusted by age, sex, BMI (except for ALI), site of onset, diagnostic delay and ALSFRS‐R score.

Abbreviations: 95% CI, 95% confidence interval; AGR, albumin‐to‐globulin ratio; ALI, advanced lung cancer inflammation index; ALS, amyotrophic lateral sclerosis; BMI, body mass index; CAR, C‐reactive protein‐to‐albumin ratio; CRP, C‐reactive protein; GLR, glucose‐to‐lymphocyte ratio; GNRI, geriatric nutritional risk index; IBI, inflammatory burden index; LCR, lymphocyte‐to‐C‐reactive protein ratio; mGNRI, modified geriatric nutritional risk index; NLR, neutrophil‐to‐lymphocyte ratio; NRI, nutritional risk index; PLR, platelet‐to‐lymphocyte ratio; PNI, prognostic nutritional index; SI, sarcopenia index; SII, systemic immune inflammation index.

We further classified SI into continuous variables, cut‐off values and interquartile variables and conducted uni‐ and multivariate Cox proportional hazards regression analyses of SI and ALS survival. After adjusting for confounders, multivariate Cox proportional hazards regression analyses revealed that the SI was an independent prognostic factor for patients with ALS. For every one standard deviation increased in the SI, the risk of poor prognosis for ALS patients decreased by 2.0% (HR, 0.98; 95% CI, 0.97–0.99; *p* < 0.001). When the SI was divided into quartiles, the lowest quartiles Q1 was used as the reference. Q2, Q3 and Q4 were positively associated with a good prognosis (*p* < 0.001). Under the model of independent effects, the HRs for survival were 0.64 (0.48–0.86), 0.49 (0.35–0.68) and 0.45 (0.32–0.64), respectively (Table 3).

**TABLE 3:** Association between SI and overall survival of patients with ALS.

SI	Model 0	*p*	Model 1	*p*	Model 2	*p*
Continuous (per SD)	0.98 (0.97–0.99)	< 0.001	0.99 (0.98–0.99)	< 0.001	0.98 (0.97–0.99)	< 0.001
Cut‐off value						
C1 (< 68.8)	1		1		1	
C2 (≥ 68.8)	0.60 (0.48–0.74)	< 0.001	0.69 (0.54–0.88)	0.002	0.59 (0.46–0.76)	< 0.001
Quartiles						
Q1 (< 59.8)	1		1		1	
Q2 (59.8–69.2)	0.71 (0.54–0.95)	0.019	0.67 (0.51–0.90)	0.007	0.64 (0.48–0.86)	0.003
Q3 (69.2–79.8)	0.54 (0.40–0.72)	< 0.001	0.58 (0.42–0.80)	0.001	0.49 (0.35–0.68)	< 0.001
Q4 (≥ 79.8)	0.48 (0.36–0.64)	< 0.001	0.55 (0.40–0.76)	< 0.001	0.45 (0.32–0.64)	< 0.001
*p* for trend		< 0.001		0.001		< 0.001

*Note:* Model 0, unadjusted model; Model 1, adjusted by age, sex and BMI; Model 2, adjusted by age, sex, BMI, site of onset, diagnostic delay and ALSFRS‐R score.

Abbreviations: ALS, amyotrophic lateral sclerosis; SD, standard deviation; SI, sarcopenia index.

### 3.3 Inflammation/Nutrition‐Based Indicators and Muscle Mass–Related Indicators and ALS Survival in Different Subgroups

A low LCR, low AGR, low creatinine and low SI were risk factors for mortality in male ALS patients, and a low PLR, high PNI, low creatinine and low SI were risk factors for mortality in female patients (Tables S2 and S3). The results showed that PLR, NLR, LCR, GLR, cystatin C and SI in patients with bulbar onset and PLR, PNI, creatinine and SI in limb onset patients were independent predictive factors for ALS survival (Tables S4 and S5). Multivariable Cox proportional hazards regression analyses confirmed that SII, PLR, AGR, PNI, GNRI, creatinine, cystatin C and SI in aged < 60 years patients and PNI, GLR and SI in patients with aged ≥ 60 years were independent predictors of survival (Tables S6 and S7). Figures S1–S6 showed the Kaplan–Meier curves of the independent predictors in different subgroups of ALS patients stratified by sex, site of onset and age.

### 3.4 The Prognostic Ability Comparison of the Inflammation/Nutrition‐Based and Muscle Mass–Related Indicators

A time‐ROC and C‐index were performed to compare the prognostic predictive capacity of inflammation/nutrition‐based and muscle mass–related indicators in patients with ALS. Compared with the other independent predictive indicators, the SI showed the highest C‐index for survival in ALS patients at 1, 3 and 5 years: 0.65 (95% CI, 0.54–0.75), 0.61 (95% CI, 0.57–0.65) and 0.59 (95% CI, 0.55–0.62), respectively (Table 4). Moreover, the SI had a higher AUC value than the other indicators (Table 5 and Figure S7).

**TABLE 4:** The time‐dependent C‐index of 17 indicators for ALS patients.

Indicators	1‐year		3‐year		5‐year	
	C‐index (95% CI)	*p*	C‐index (95% CI)	*p*	C‐index (95% CI)	*p*
ALI	0.55 (0.42–0.69)	0.351	0.53 (0.49–0.58)	0.019	0.53 (0.50–0.57)	0.036
SII	0.53 (0.38–0.67)	0.225	0.50 (0.45–0.55)	< 0.001	0.51 (0.47–0.54)	0.002
PLR	0.52 (0.38–0.66)	0.161	0.52 (0.47–0.56)	0.005	0.52 (0.48–0.55)	0.005
NLR	0.55 (0.41–0.69)	0.354	0.52 (0.47–0.57)	0.006	0.51 (0.48–0.55)	0.004
CRP	0.57 (0.43–0.71)	0.297	0.52 (0.47–0.57)	0.004	0.51 (0.47–0.55)	0.003
CAR	0.59 (0.43–0.75)	0.431	0.53 (0.48–0.58)	0.009	0.52 (0.48–0.56)	0.005
LCR	0.57 (0.42–0.72)	0.322	0.52 (0.47–0.57)	0.004	0.52 (0.48–0.56)	0.004
AGR	0.71 (0.59–0.83)	0.431	0.53 (0.48–0.58)	0.008	0.53 (0.50–0.57)	0.018
IBI	0.55 (0.40–0.70)	0.250	0.52 (0.47–0.57)	0.006	0.52 (0.48–0.56)	0.007
PNI	0.57 (0.44–0.70)	0.453	0.51 (0.47–0.56)	0.004	0.53 (0.49–0.56)	0.015
GLR	0.54 (0.41–0.68)	0.311	0.51 (0.46–0.56)	0.003	0.51 (0.47–0.54)	0.002
GNRI	0.56 (0.46–0.65)	0.226	0.54 (0.49–0.58)	0.015	0.54 (0.51–0.58)	0.058
mGNRI	0.58 (0.45–0.71)	0.326	0.51 (0.46–0.56)	0.001	0.50 (0.46–0.54)	<0.001
NRI	0.56 (0.46–0.65)	0.232	0.54 (0.49–0.58)	0.015	0.54 (0.51–0.58)	0.057
Creatinine	0.52 (0.40–0.64)	0.067	0.54 (0.49–0.58)	< 0.001	0.53 (0.49–0.56)	< 0.001
Cystatin C	0.64 (0.50–0.78)	0.906	0.60 (0.55–0.64)	0.565	0.58 (0.54–0.62)	0.663
SI	0.65 (0.54–0.75)	NA	0.61 (0.57–0.65)	NA	0.59 (0.55–0.62)	NA

*Note:* The *p* value indicates the difference between the other indicators and SI.

Abbreviations: AGR, albumin‐to‐globulin ratio; ALI, advanced lung cancer inflammation index; ALS, amyotrophic lateral sclerosis; CAR, C‐reactive protein‐to‐albumin ratio; CRP, C‐reactive protein; GLR, glucose‐to‐lymphocyte ratio; GNRI, geriatric nutritional risk index; IBI, inflammatory burden index; LCR, lymphocyte‐to‐C‐reactive protein ratio; mGNRI, modified geriatric nutritional risk index; NLR, neutrophil‐to‐lymphocyte ratio; NRI, nutritional risk index; PLR, platelet‐to‐lymphocyte ratio; PNI, prognostic nutritional index; SI, sarcopenia index; SII, systemic immune inflammation index.

**TABLE 5:** The time‐dependent ROC of 17 indicators in patients with ALS.

Indicators	1‐year		3‐year		5‐year	
	AUC (95% CI)	*p*	AUC (95% CI)	*p*	AUC (95% CI)	*p*
ALI	0.56 (0.42–0.69)	0.377	0.54 (0.48–0.59)	0.016	0.54 (0.48–0.60)	0.095
SII	0.53 (0.38–0.67)	0.223	0.51 (0.45–0.56)	0.003	0.56 (0.50–0.62)	0.309
PLR	0.52 (0.38–0.66)	0.166	0.54 (0.48–0.59)	0.024	0.56 (0.50–0.61)	0.253
NLR	0.55 (0.41–0.69)	0.367	0.52 (0.46–0.57)	0.003	0.51 (0.45–0.57)	0.029
CRP	0.58 (0.43–0.72)	0.321	0.52 (0.46–0.58)	0.003	0.57 (0.50–0.63)	0.230
CAR	0.59 (0.43–0.75)	0.441	0.52 (0.46–0.58)	0.006	0.56 (0.49–0.62)	0.163
LCR	0.57 (0.41–0.72)	0.332	0.51 (0.45–0.57)	0.003	0.57 (0.50–0.63)	0.235
AGR	0.71 (0.59–0.83)	0.444	0.53 (0.47–0.58)	0.004	0.53 (0.47–0.59)	0.041
IBI	0.56 (0.40–0.71)	0.267	0.52 (0.46–0.58)	0.007	0.58 (0.51–0.64)	0.339
PNI	0.58 (0.44–0.71)	0.485	0.51 (0.45–0.56)	0.001	0.53 (0.48–0.59)	0.063
GLR	0.54 (0.41–0.68)	0.331	0.51 (0.50–0.53)	<0.001	0.51 (0.45–0.57)	0.023
GNRI	0.56 (0.46–0.66)	0.248	0.55 (0.50–0.61)	0.034	0.57 (0.51–0.63)	0.325
mGNRI	0.59 (0.46–0.72)	0.351	0.50 (0.44–0.57)	0.002	0.53 (0.47–0.59)	0.047
NRI	0.56 (0.46–0.66)	0.253	0.55 (0.50–0.61)	0.034	0.57 (0.51–0.63)	0.322
Creatinine	0.52 (0.40–0.65)	0.082	0.55 (0.50–0.61)	0.002	0.54 (0.48–0.60)	0.021
Cystatin C	0.64 (0.49–0.78)	0.901	0.61 (0.56–0.66)	0.557	0.58 (0.52–0.64)	0.524
SI	0.65 (0.54–0.76)	NA	0.63 (0.58–0.68)	NA	0.60 (0.55–0.66)	NA

*Note:* The *p* value indicates the difference between the other indicators and SI.

Abbreviations: AGR, albumin‐to‐globulin ratio; ALI, advanced lung cancer inflammation index; ALS, amyotrophic lateral sclerosis; AUC, area under curve; CAR, C‐reactive protein‐to‐albumin ratio; CRP, C‐reactive protein; GLR, glucose‐to‐lymphocyte ratio; GNRI, geriatric nutritional risk index; IBI, inflammatory burden index; LCR, lymphocyte‐to‐C‐reactive protein ratio; mGNRI, modified geriatric nutritional risk index; NLR, neutrophil‐to‐lymphocyte ratio; NRI, nutritional risk index; PLR, platelet‐to‐lymphocyte ratio; PNI, prognostic nutritional index; ROC, receiver operating characteristic; SI, sarcopenia index; SII, systemic immune inflammation index.

In different subgroups, the SI had the highest C‐index in men, women, limb onset and aged < 60 years ALS patients, as compared with the other independent predictive indicators (Tables S8–S10). In the subgroups of bulbar onset and aged ≥ 60 years, cystatin C and PNI had the highest C‐index compared to the other independent predictive indicators, separately (Tables S8–S10).

### 3.5 Randomized Internal Validation of the Association Between SI and Survival in ALS Patients

Subsequently, we randomly assigned the total participants to validation cohorts A (304 cases) and B (202 cases), with a 6:4 ratio based on computer‐generated random numbers (Table S11). SI was an independent predictive factor for ALS patients in both validation cohorts A (HR, 0.98; 95% CI, 0.97–0.99; *p* < 0.001) and B (HR, 0.98; 95% CI, 0.96–0.99; *p* = 0.001) (Table 6).

**TABLE 6:** Association between SI and overall survival of patients with ALS at validation cohorts.

SI	Model 0	*p*	Model 1	*p*	Model 2	*p*
Validation cohort A						
Continuous (per SD)	0.98 (0.97–0.99)	< 0.001	0.98 (0.97–0.99)	0.001	0.98 (0.97–0.99)	< 0.001
Cut‐off value						
C1 (< 68.8)	1		1		1	
C2 (≥ 68.8)	0.59 (0.45–0.77)	< 0.001	0.64 (0.47–0.87)	0.005	0.63 (0.46–0.87)	0.004
Quartiles						
Q1 (< 59.0)	1		1		1	
Q2 (59.0–69.1)	0.85 (0.59–1.21)	0.359	/	0.595	0.71 (0.49–1.01)	0.058
Q3 (69.1–78.9)	0.55 (0.37–0.80)	0.002	/	0.185	0.52 (0.34–0.80)	0.003
Q4 (≥ 78.9)	0.52 (0.35–0.75)	0.001	/	0.208	0.49 (0.32–0.75)	0.001
*p* for trend		0.001		0.084		0.006
Validation cohort B						
Continuous (per SD)	0.98 (0.97–0.99)	0.004	/	0.166	0.98 (0.96–0.99)	0.001
Cut‐off value						
C1 (< 68.8)	1		1		1	
C2 (≥ 68.8)	0.60 (0.42–0.85)	0.004	/	0.218	0.55 (0.37–0.82)	0.003
Quartiles						
Q1 (< 61.4)	1		1		1	
Q2 (61.4–69.5)	0.76 (0.47–1.22)	0.252	/	0.750	/	0.945
Q3 (69.5–80.6)	0.65 (0.40–1.04)	0.074	/	0.925	/	0.583
Q4 (≥ 80.6)	0.47 (0.29–0.77)	0.003	/	0.248	/	0.252
*p* for trend		0.020		0.484		0.331

*Note:* Model 0, unadjusted model; Model 1, adjusted by age, sex and BMI; Model 2, adjusted by age, sex, BMI, site of onset, diagnostic delay and ALSFRS‐R score.

Abbreviations: ALS, amyotrophic lateral sclerosis; SD, standard deviation; SI, sarcopenia index.

## 4 Discussion

Increasing evidence suggested that inflammation/nutrition‐based and muscle mass–related indicators can be reliable prognostic predictors in patients with wasting disease. However, the optimal predictor for ALS patients remains unclear. Here, we constructed a large cohort, evaluated 17 inflammation/nutrition‐based and muscle mass–related indicators and found that SI is a stable and consistent prognostic predictor in all ALS patients and most subgroups. Additionally, cystatin C emerges as a superior indicator in the subgroup of patients with bulbar onset, whereas PNI proves to be a preferable prognostic predictor for patients aged ≥ 60 years.

Previous studies have shown that SII [14], CRP [12, 24], NLR [13], creatinine [16] and cystatin C [21] were prognostic predictors in ALS patients, whereas PLR [14] was not associated with survival. In alignment with these studies, we found SII, NLR (for patients with bulbar onset), creatinine and cystatin C (for patients with bulbar onset and aged < 60 years) are independent predictors of survival in ALS patients. Contrary to prior research, our study reveals PLR to be an independent prognostic predictor, whereas CRP levels are not associated with ALS survival. Markers such as ALI, CAR, LCR, IBI, AGR, PNI, GLR, GNRI, mGNRI, NRI and SI have not been extensively studied before. Our findings suggested that mGNRI, SI, LCR (for men and bulbar onset), AGR (for men and aged < 60 years), PNI (for women, limb onset, aged < 60 years and aged ≥ 60 years), GLR (for bulbar onset and aged ≥ 60 years) and GNRI (for aged < 60 years) are associated with survival in ALS patients. Among the 17 inflammation/nutrition‐based and muscle mass–related indicators, SI emerges as the optimal prognostic predictor. SI can be an objective indicator, easy to detect and use, reflecting ALS residual muscle mass and facilitating timely prediction of patient outcomes in clinical practice.

The dysregulation of the immune system plays a multifaceted role in ALS [14]. SII, NLR and PLR are the markers of innate immunity [14]. Consequently, these three indicators were identified as independent prognostic predictors.

Albumin and globulin, the primary components of serum protein, play a vital role in the systemic inflammatory response. LCR reflects systemic inflammatory status, and AGR and LCR have been identified as prognostic factors. The systemic environment of ALS circulation is predominantly pro‐inflammatory, characterized by increased lipid peroxides and oxidative stress, pro‐inflammatory monocytes/macrophages and cytokines, increased neurotoxic NK cells, dysfunctional Tregs and altered gut microbiota. Moreover, the widespread dysregulation of immune cells and cytokines in ALS patients reflects an increased disease burden and accelerated progression, reinforcing the notion of ALS as a disease with an extensive systemic proinflammatory response [25]. GNRI is a predictor of nutrition‐related complications and muscle dysfunction in the elderly [26, 27]. Given its utility in this population, GNRI is also suitable for assessing the nutritional status of ALS patients, who face comparable challenges with chewing and swallowing.

mGNRI, as a combined indicator of systemic inflammation and nutrition, holds considerable potential for prognostic assessment in cancer patients [28]. In previous studies, CRP, a biomarker of the inflammatory response, has shown significant prognostic value in ALS patients [12, 24]. Although CRP was not identified to be significantly associated with ALS survival in our study, the mGNRI, which combines CRP and nutritional status, emerged as an independent prognostic predictor. This suggested that the combination of inflammation and nutrition is a sensitive predictor. Similarly, PNI and GLR, which are combined indicators of nutrition and immune status, showed promise as sensitive prognostic predictors.

Here, we found that ALI, CAR, IBI and NRI were not related to ALS survival. ALI is known for comprehensively assessing systemic inflammation and metabolic status, especially in cancer [29]. CAR reflects potential inflammatory and nutritional status [30], whereas IBI, an index to assess the inflammatory burden, is a powerful prognostic indicator for cancer patients [31]. NRI is employed as an indicator of nutritional status [32]. Despite the presence of malnutrition and inflammatory status in ALS patients, we did not find significant associations between factors assessing nutrition and/or inflammation, including ALI, CAR, IBI and NRI, with survival, indicating that they are not sensitive or suitable predictors.

An important finding of our study is that the SI predicts survival with the highest C‐index and AUC value across all ALS patients and most subgroups, suggesting its superior predictive efficacy among the 17 inflammation/nutrition‐based and muscle mass–related indicators. Kashani et al. validated the correlation between the creatinine‐to‐cystatin C ratio and muscle mass and defined it as a sarcopenia index in 2017 [22]. Several studies confirmed that the creatinine‐to‐cystatin C ratio correlates with CT‐derived and dual‐energy x‐ray absorptiometry‐derived skeletal muscle mass [33, 34]. Serum creatinine and cystatin C are widely used to evaluate renal function. Serum creatinine, primarily produced during skeletal muscle metabolism from creatine phosphate, varies with muscle mass and is lower in patients with reduced muscle mass [35]. Meanwhile, serum cystatin C, a non‐ionic protein produced at a constant rate by nucleated cells, is unaffected by muscle metabolism [36]. Based on these features, SI can better assess muscle mass and show potential for screening for sarcopenia. Its value has been successively verified in critically ill patients [37], the elderly [38], organ transplant patients [39] and type 2 diabetes patients [40].

Serum creatinine is produced at a fairly constant rate by the body and has been shown to correlate with lean body mass in healthy individuals [41] and in adults and children with various diseases [42, 43, 44]. However, serum creatinine levels are affected by renal function. By calculating the serum SI, we eliminated the effect of renal function on serum creatinine. In particular, cystatin C was found to be an independent predictor of ALS in our previous studies [21] and by expanding the sample size in this study, the results showed that cystatin C is an independent prognostic factor only in the bulbar onset and aged < 60 year subgroups. Both these two studies indicated that creatinine is a predictor of ALS survival. Additionally, higher creatinine and lower cystatin C levels predict longer survival in ALS patients. Consequently, the ratio of creatinine to cystatin C (i.e., the sarcopenia index) might have higher predictive efficiency. SI reflects the muscle mass status of the entire body. Therefore, we recommend measuring serum SI to predict prognosis in ALS patients. Furthermore, serum SI is more convenient for clinical use and facilitates better adherence to monitoring disease status and progression in ALS patients.

We have to acknowledge several limitations in this study. First, there was an absence of data to measure actual residual muscle mass. However, determining total residual muscle mass in ALS patients is inherently challenging. Thus, based on previous studies and our findings, it is reasonable to suggest that serum SI may serve as a surrogate indicator of residual muscle mass to predict survival. Second, this study was conducted at a single centre, and multicentre prospective cohort studies are necessary to further validate our findings. Third, the 17 indicators were measured at baseline, but changes in these indicators during follow‐up were absent in our study. Although we followed up on the prognosis of ALS patients, allowing us to analyse the association between the 17 indicators and survival using the Cox model, dynamic observation of these indicators as the disease progresses is critical. We will continue to complete the dynamic monitoring of these indicators in future studies. Fourth, ALS patients were advised to use riluzole and/or edaravone, but not all patients received these treatments due to practical limitations. Given the benefits of riluzole and edaravone, their varied administration could impact the study results. However, previous studies showed that riluzole and edaravone can only modestly extend the survival [45, 46]. Additionally, the proportion of riluzole/edaravone use was similar in the high and low groups of different indicators, suggesting that the influence of medication history on survival time might be very limited. Finally, we followed ALS patients hospitalized from January 2014 to December 2019 and registered their basic demographic information. The initial aim of this cohort study was to observe the survival of ALS patients, and therefore, some of the haematological data were incomplete. Of the 789 ALS patients initially recruited, only 506 were finally included, with 35.8% excluded due to exclusion criteria and missing haematological data, implying a potential selection bias. However, we analysed the demographic characteristics of both the 789 initially recruited patients and the 506 enrolled patients and found no significant difference in characteristics such as sex, age and site of onset (Table S12).

In conclusion, SI demonstrated superior performance in predicting the prognosis of ALS patients overall and in most subgroups compared to other indicators. Evaluating SI could help identify ALS patients with a poor prognosis. Given its simplicity and availability, serum SI is well suited for clinical use in assessing the prognosis of ALS patients.

## Ethics Statement

This study was conducted in compliance with the Declaration of Helsinki and approved by the Ethics Committee of Chinese PLA General Hospital. Written informed consent was obtained from participants.

## Conflicts of Interest

The authors declare no conflicts of interest.

## Supporting information


**Figure S1** The Kaplan–Meier curves of the LCR (a), AGR (b), Creatinine (c) and SI (d) in male patients with amyotrophic lateral sclerosis. LCR, lymphocyte‐to‐C‐reactive protein ratio; AGR, albumin‐to‐globulin ratio; SI, sarcopenia index.

**Figure S2** The Kaplan–Meier curves of the PLR (a), PNI (b), Creatinine (c) and SI (d) in female patients with amyotrophic lateral sclerosis. PLR, platelet‐to‐lymphocyte ratio; PNI, prognostic nutritional index; SI, sarcopenia index.

**Figure S3** The Kaplan–Meier curves of the PLR (a), NLR (b), LCR (c), GLR (d), Cystatin C (e) and SI (f) in patients with bulbar onset. PLR, platelet‐to‐lymphocyte ratio; NLR, neutrophil‐to‐lymphocyte ratio; LCR, lymphocyte‐to‐C‐reactive protein ratio; GLR, glucose‐to‐lymphocyte ratio; SI, sarcopenia index.

**Figure S4** The Kaplan–Meier curves of the PLR (a), PNI (b), Creatinine (c) and SI (d) in patients with limb onset. PLR, platelet‐to‐lymphocyte ratio; PNI, prognostic nutritional index; SI, sarcopenia index.

**Figure S5** The Kaplan–Meier curves of the SII (a), PLR (b), AGR (c), PNI (d), GNRI (e), Creatinine (f), Cystatin C (g) and SI (h) in patients aged<60 years. SII, systemic immune‐inflammation index; PLR, platelet‐to‐lymphocyte ratio; AGR, albumin‐to‐globulin ratio; PNI, prognostic nutritional index; GNRI, geriatric nutritional risk index; SI, sarcopenia index.

**Figure S6** The Kaplan–Meier curves of the PNI (a), GLR (b), and SI (c) in patients aged**≥**60 years. PLR, platelet‐to‐lymphocyte ratio; GLR, glucose‐to‐lymphocyte ratio; SI, sarcopenia index.

**Figure S7** The time‐dependent ROC in patients with amyotrophic lateral sclerosis of SI. ROC: receiver operating characteristic; AUC: area under curve; SI, sarcopenia index.

**Table S1** Calculation methods of combination in each nutrition/inflammation‐based and muscle mass‐related indicator.
**Table S2.** Univariate and Multivariate Cox survival analysis in male patients.
**Table S3.** Univariate and Multivariate Cox survival analysis in female patients.
**Table S4.** Univariate and Multivariate Cox survival analysis in patients with bulbar onset.
**Table S5.** Univariate and Multivariate Cox survival analysis in patients with limb onset.
**Table S6.** Univariate and Multivariate Cox survival analysis in patients aged<60 years.
**Table S7.** Univariate and Multivariate Cox survival analysis in patients aged≥60 years.
**Table S8.** The C‐index of 17 indicators for ALS patients stratified by sex.
**Table S9.** The C‐index of 17 indicators for ALS patients stratified by site of onset.
**Table S10.** The C‐index of 17 indicators for ALS patients stratified by age.
**Table S11.** Demographics characteristics of ALS patients between validation cohorts A and validation cohorts B.
**Table S12.** Demographic characteristics of the finally included ALS patients and the initially screened ALS patients
