# Supplementary figures and images for: Prognostic Value of Systemic Inflammation, Nutritional Status and Sarcopenia in Patients With Amyotrophic Lateral Sclerosis
Source: J Cachexia Sarcopenia Muscle. 2024 Oct 24;15(6):2743–55. doi: 10.1002/jcsm.13618 (PMC11634485; doi:10.1002/jcsm.13618)

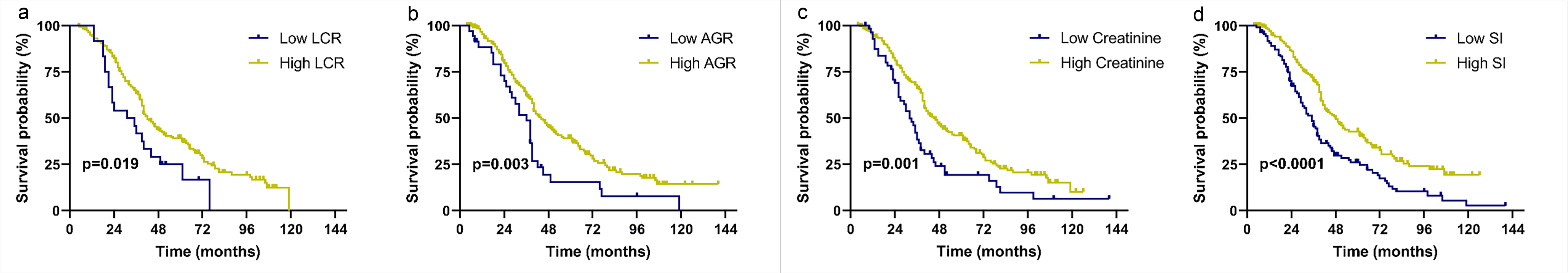

Supplement: Supplementary file 1 — Figure S1 The Kaplan–Meier curves of the LCR (a), AGR (b), Creatinine (c) and SI (d) in male patients with amyotrophic lateral sclerosis. LCR, lymphocyte‐to‐C‐reactive protein ratio; AGR, albumin‐to‐globulin ratio; SI, sarcopenia index. [file JCSM-15-2743-s003.tif]

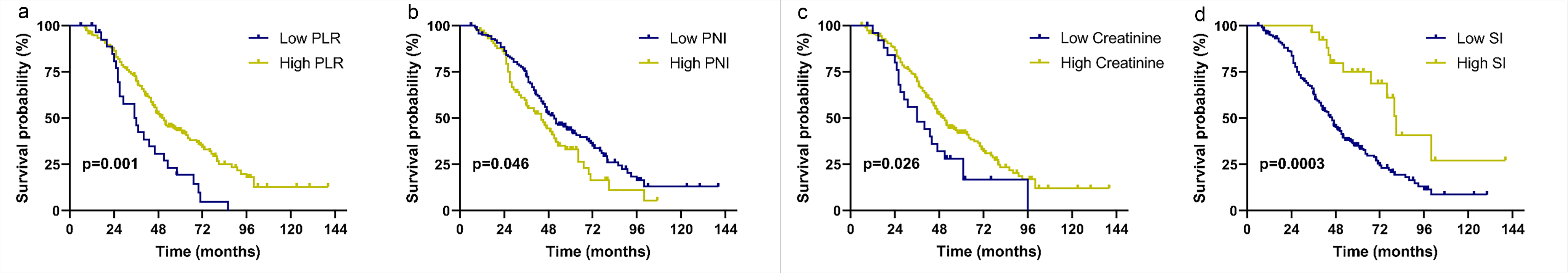

Supplement: Supplementary file 2 — Figure S2 The Kaplan–Meier curves of the PLR (a), PNI (b), Creatinine (c) and SI (d) in female patients with amyotrophic lateral sclerosis. PLR, platelet‐to‐lymphocyte ratio; PNI, prognostic nutritional index; SI, sarcopenia index. [file JCSM-15-2743-s006.tif]

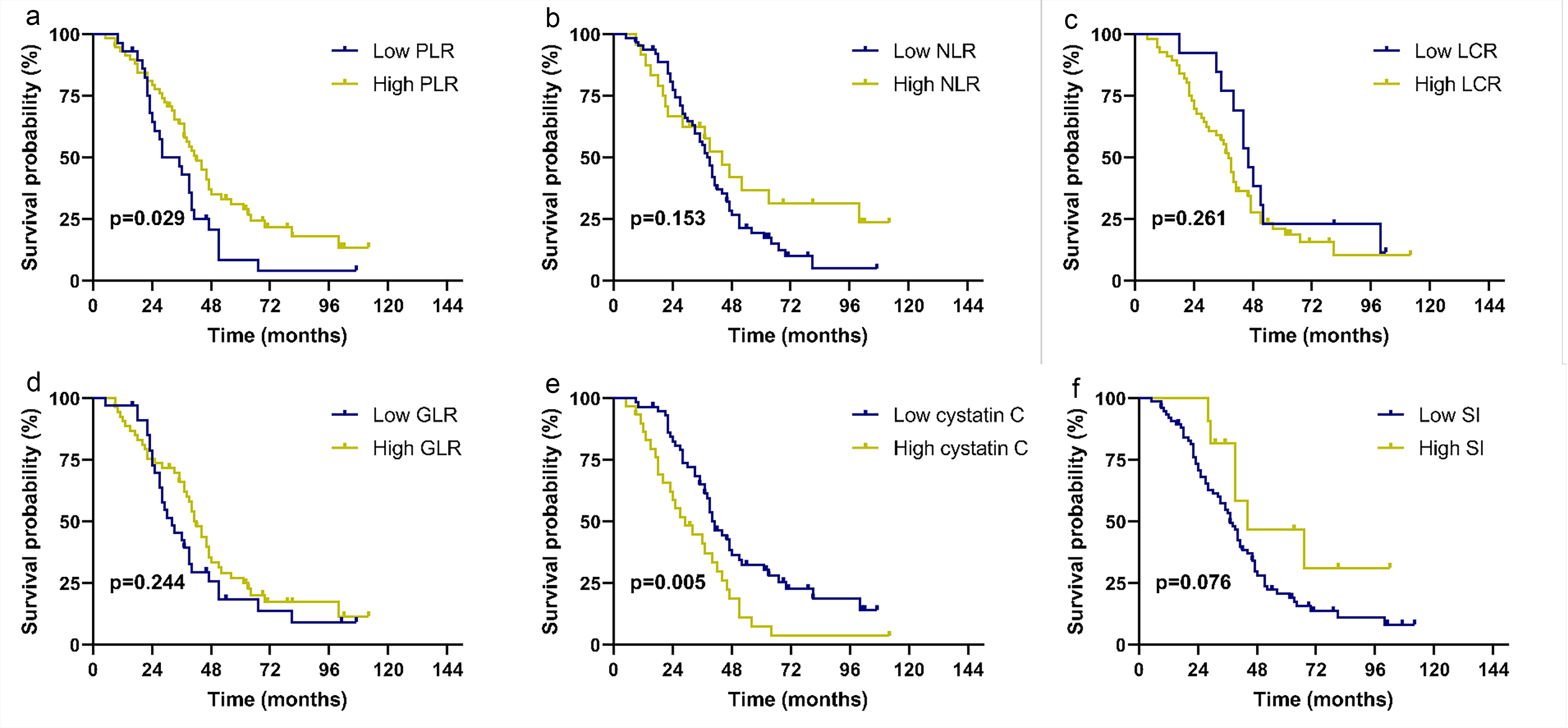

Supplement: Supplementary file 3 — Figure S3 The Kaplan–Meier curves of the PLR (a), NLR (b), LCR (c), GLR (d), Cystatin C (e) and SI (f) in patients with bulbar onset. PLR, platelet‐to‐lymphocyte ratio; NLR, neutrophil‐to‐lymphocyte ratio; LCR, lymphocyte‐to‐C‐reactive protein ratio; GLR, glucose‐to‐lymphocyte ratio; SI, sarcopenia index. [file JCSM-15-2743-s008.tif]

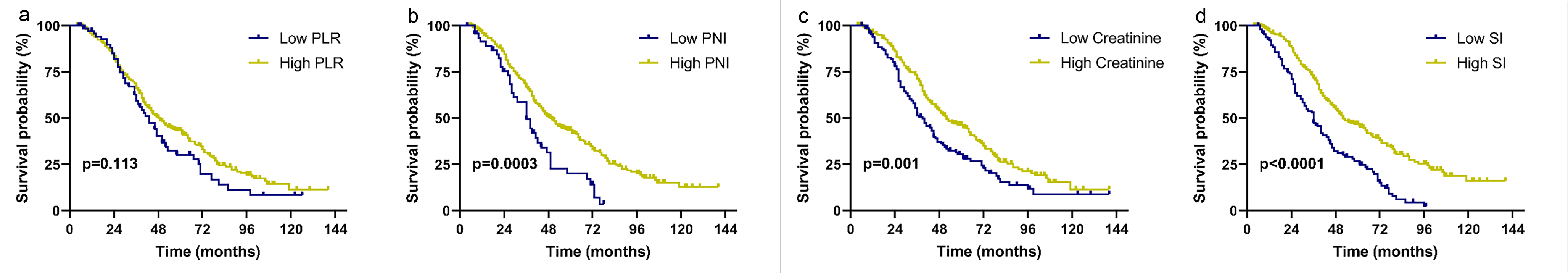

Supplement: Supplementary file 4 — Figure S4 The Kaplan–Meier curves of the PLR (a), PNI (b), Creatinine (c) and SI (d) in patients with limb onset. PLR, platelet‐to‐lymphocyte ratio; PNI, prognostic nutritional index; SI, sarcopenia index. [file JCSM-15-2743-s002.tif]

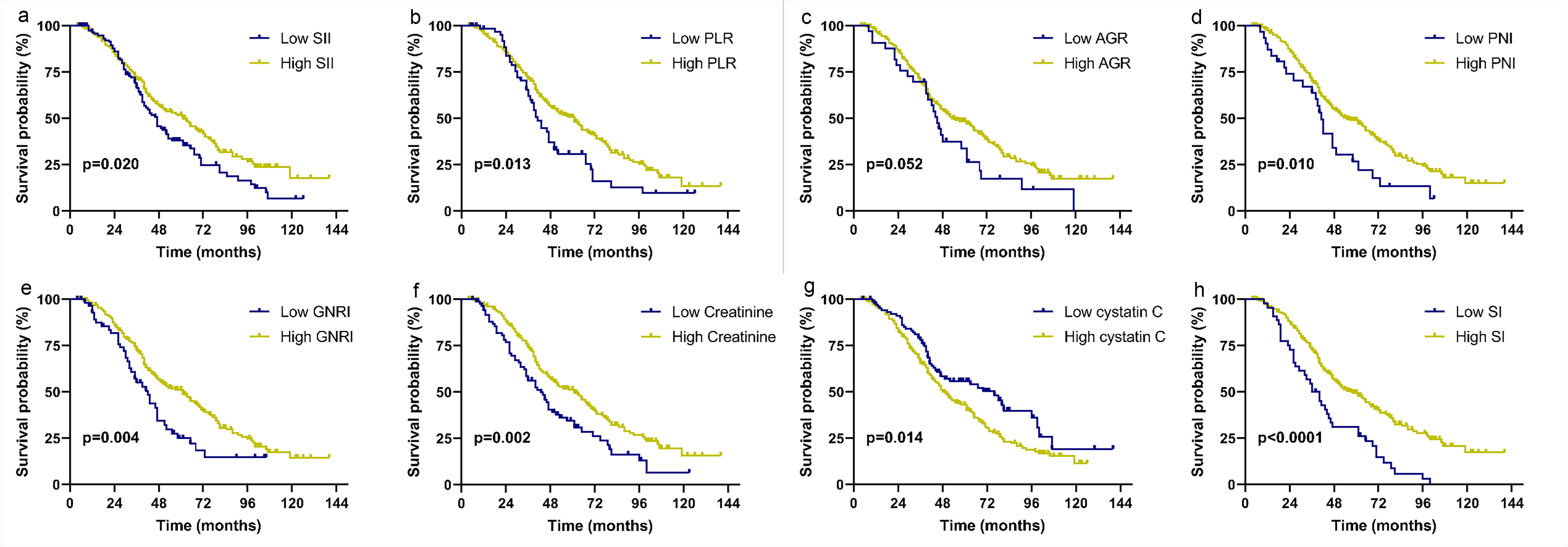

Supplement: Supplementary file 5 — Figure S5 The Kaplan–Meier curves of the SII (a), PLR (b), AGR (c), PNI (d), GNRI (e), Creatinine (f), Cystatin C (g) and SI (h) in patients aged<60 years. SII, systemic immune‐inflammation index; PLR, platelet‐to‐lymphocyte ratio; AGR, albumin‐to‐globulin ratio; PNI, prognostic nutritional index; GNRI, geriatric nutritional risk index; SI, sarcopenia index. [file JCSM-15-2743-s001.tif]

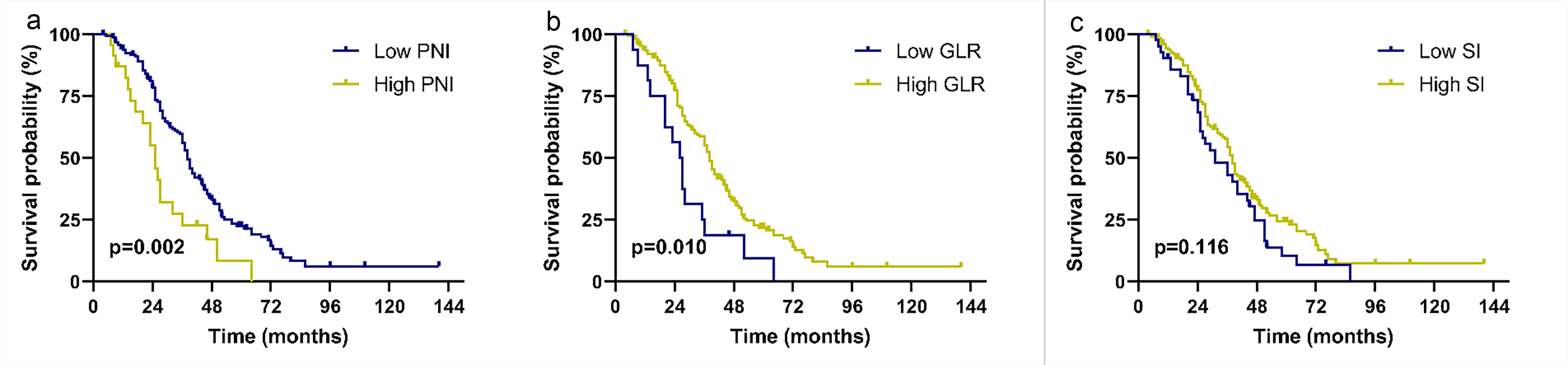

Supplement: Supplementary file 6 — Figure S6 The Kaplan–Meier curves of the PNI (a), GLR (b), and SI (c) in patients aged≥60 years. PLR, platelet‐to‐lymphocyte ratio; GLR, glucose‐to‐lymphocyte ratio; SI, sarcopenia index. [file JCSM-15-2743-s007.tif]

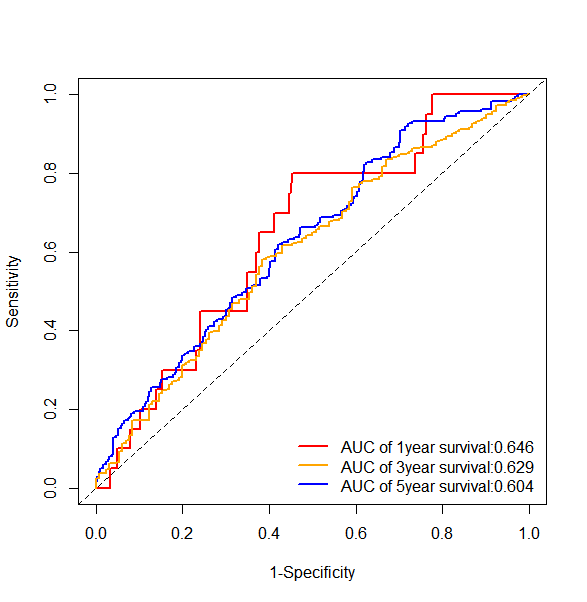

Supplement: Supplementary file 7 — Figure S7 The time‐dependent ROC in patients with amyotrophic lateral sclerosis of SI. ROC: receiver operating characteristic; AUC: area under curve; SI, sarcopenia index. [file JCSM-15-2743-s005.tiff]
