# Supplementary material for: Prognostic Value of Systemic Inflammation, Nutritional Status and Sarcopenia in Patients With Amyotrophic Lateral Sclerosis
Source: J Cachexia Sarcopenia Muscle. 2024 Oct 24;15(6):2743–55. doi: 10.1002/jcsm.13618 (PMC11634485; doi:10.1002/jcsm.13618)
Supplement: Supplementary file 8 — Table S1 Calculation methods of combination in each nutrition/inflammation‐based and muscle mass‐related indicator. Table S2. Univariate and Multivariate Cox survival analysis in male patients. Table S3. Univariate and Multivariate Cox survival analysis in female patients. Table S4. Univariate and Multivariate Cox survival analysis in patients with bulbar onset. Table S5. Univariate and Multivariate Cox survival analysis in patients with limb onset. Table S6. Univariate and Multivariate Cox survival analysis in patients aged<60 years. Table S7. Univariate and Multivariate Cox survival analysis in patients aged≥60 years. Table S8. The C‐index of 17 indicators for ALS patients stratified by sex. Table S9. The C‐index of 17 indicators for ALS patients stratified by site of onset. Table S10. The C‐index of 17 indicators for ALS patients stratified by age. Table S11. Demographics characteristics of ALS patients between validation cohorts A and validation cohorts B. Table S12. Demographic characteristics of the finally included ALS patients and the initially screened ALS patients [file JCSM-15-2743-s004.doc]

**Table S1.** **Calculation methods of combination in each nutrition/inflammation-based and muscle mass-related indicator.**

| **Indicators** | **Definition or calculation formula** |
| --- | --- |
| **ALI** | BMI (kg/m^2^) × albumin (g/dl)/NLR (×10^9^) |
| **SII** | platelet count (×10^9^) × neutrophil count (×10^9^)/lymphocyte count (×10^9^) |
| **PLR** | platelet count (×10^9^)/lymphocyte count (×10^9^) |
| **NLR** | neutrophil count (×10^9^)/ lymphocyte count (×10^9^) |
| **CAR** | C-reactive protein (mg/L)/ albumin (g/L) |
| **LCR** | 10,000×lymphocyte count (×10^9^)/CRP (mg/L) |
| **IBI** | C-reactive protein (mg/dL)×neutrophil (/μL)/lymphocyte (/μL) |
| **AGR** | albumin (g/L)/globulin (g/L) |
| **PNI** | albumin (g/L) + 5×lymphocyte count (×10^9^) |
| **GLR** | glucose (mmol/l)/ lymphocyte count (×10^9^) |
| **GNRI** | 1.489×albumin (g/L) + (41.7× current weight/ ideal body weight (IBW) |
| **mGNRI** | 14.89/CRP (mg/L)+41.7× current weight/ IBW |
| **NRI** | 1.519×albumin (g/L) + 41.7× current weight/ IBW |
| **SI** | 100×creatinine (mg/dL)/cystatin C (mg/L) |

If current weight was greater than IBW, current/IBW was regarded as 1. The IBW of men and women were calculated by the formula, height(cm)-100-[height (cm)-150]/4 and height(cm)-100-[height (cm)-150]/2.5, respectively.

**Table S2. Univariate and Multivariate Cox survival analysis in male patients.**

|  |  | Model 0 | | Model 1 | | Model 2 | |
| --- | --- | --- | --- | --- | --- | --- | --- |
| Variables | Number of patients | Hazard Ratio (95% CI) | p-value | Hazard Ratio (95% CI) | p-value | Hazard Ratio (95% CI) | p-value |
| ALI |  |  |  |  |  |  |  |
| <59.76 | 175 | 1 | 0.027 | / | 0.512 | / | 0.674 |
| ≥59.76 | 113 | 0.72 (0.54-0.96) |  | / |  | / |  |
| SII |  |  |  |  |  |  |  |
| <541.56 | 229 | 1 | 0.165 | / | 0.499 | / | 0.857 |
| ≥541.56 | 59 | 1.27 (0.91-1.79) |  | / |  | / |  |
| PLR |  |  |  |  |  |  |  |
| <111.47 | 137 | 1 | 0.027 | / | 0.112 | / | 0.149 |
| ≥111.47 | 151 | 1.38 (1.04-1.82) |  | / |  | / |  |
| NLR |  |  |  |  |  |  |  |
| <1.34 | 57 | 1 | 0.395 | / | 0.969 | / | 0.520 |
| ≥1.34 | 231 | 1.17 (0.82-1.66) |  | / |  | / |  |
| CRP |  |  |  |  |  |  |  |
| <0.74 | 55 | 1 | 0.298 | / | 0.294 | / | 0.360 |
| ≥0.74 | 233 | 1.35 (0.77-2.39) |  | / |  | / |  |
| CAR |  |  |  |  |  |  |  |
| <0.024 | 89 | 1 | 0.148 | / | 0.358 | / | 0.277 |
| ≥0.024 | 199 | 1.33 (0.90-1.97) |  | / |  | / |  |
| LCR |  |  |  |  |  |  |  |
| <4121.04 | 52 | 1 | 0.022 | / | 0.635 | 1 | 0.033 |
| ≥4121.04 | 236 | 0.58 (0.36-0.92) |  | / |  | 0.59 (0.36-0.96) |  |
| IBI |  |  |  |  |  |  |  |
| <0.36 | 162 | 1 | 0.416 | / | 0.910 | / | 0.901 |
| ≥0.36 | 126 | 1.14 (0.83-1.56) |  | / |  | / |  |
| AGR |  |  |  |  |  |  |  |
| <1.49 | 35 | 1 | 0.004 | / | 0.247 | 1 | 0.032 |
| ≥1.49 | 253 | 0.56 (0.38-0.83) |  | / |  | 0.63 (0.41-0.96) |  |
| PNI |  |  |  |  |  |  |  |
| <47.60 | 60 | 1 | 0.005 | / | 0.725 | / | 0.749 |
| ≥47.60 | 228 | 0.62 (0.44-0.87) |  | / |  | / |  |
| GLR |  |  |  |  |  |  |  |
| <2.68 | 151 | 1 | 0.031 | / | 0.214 | / | 0.430 |
| ≥2.68 | 137 | 1.36 (1.03-1.80) |  | / |  | / |  |
| GNRI |  |  |  |  |  |  |  |
| <105.28 | 204 | 1 | 0.014 | / | 0.348 | / | 0.818 |
| ≥105.28 | 84 | 0.67 (0.49-0.92) |  | / |  | / |  |
| mGNRI |  |  |  |  |  |  |  |
| <43.36 | 55 | 1 | 0.054 | / | 0.815 | / | 0.081 |
| ≥43.36 | 233 | 0.65 (0.42-1.01) |  | / |  | / |  |
| NRI |  |  |  |  |  |  |  |
| <106.56 | 204 | 1 | 0.014 | / | 0.348 | / | 0.818 |
| ≥106.56 | 84 | 0.67 (0.49-0.92) |  | / |  | / |  |
| Creatinine |  |  |  |  |  |  |  |
| <57.1 | 57 | 1 | 0.002 | 1 | 0.014 | 1 | 0.007 |
| ≥57.1 | 231 | 0.58 (0.42-0.81) |  | 0.66 (0.47-0.92) |  | 0.63 (0.45-0.88) |  |
| Cystatin C |  |  |  |  |  |  |  |
| <1.23 | 247 | 1 | 0.004 | / | 0.135 | / | 0.271 |
| ≥1.23 | 41 | 1.72 (1.19-2.48) |  | / |  | / |  |
| SI |  |  |  |  |  |  |  |
| <69.1 | 111 | 1 | 6.3×10^-5^ | 1 | 0.027 | 1 | 0.001 |
| ≥69.1 | 177 | 0.56 (0.42-0.75) |  | 0.71 (0.52-0.96) |  | 0.58 (0.42-0.80) |  |

Model 0, unadjusted model; Model 1, adjusted by age and BMI (except for ALI); Model 2, adjusted by age, BMI (except for ALI), site of onset, diagnostic delay, and ALSFRS-R score.

95% CI, 95% confidence interval; AGR, albumin‐to‐globulin ratio; ALI, advanced lung cancer inflammation index; ALS, amyotrophic lateral sclerosis; BMI, body mass index; CAR, C‐reactive protein‐to‐albumin ratio; CRP: C‐reactive protein; GLR, glucose‐to‐lymphocyte ratio; GNRI, geriatric nutritional risk index; IBI: inflammatory burden index; LCR, lymphocyte‐to‐C‐reactive protein ratio; mGNRI, modified geriatric nutritional risk index; NLR, neutrophil‐to‐lymphocyte ratio; NRI, nutritional risk index; PLR, platelet‐to‐lymphocyte ratio; PNI, prognostic nutritional index; SI, sarcopenia index; SII, systemic immune‐inflammation index.

**Table S3. Univariate and Multivariate Cox survival analysis in female patients.**

|  |  | Model 0 | | Model 1 | | Model 2 | |
| --- | --- | --- | --- | --- | --- | --- | --- |
| Variables | Number of patients | Hazard Ratio (95% CI) | p-value | Hazard Ratio (95% CI) | p-value | Hazard Ratio (95% CI) | p-value |
| ALI |  |  |  |  |  |  |  |
| <75.08 | 166 | 1 | 0.022 | 1 | 0.025 | / | 0.069 |
| ≥75.08 | 52 | 1.54 (1.07-2.23) |  | 1.53 (1.06-2.21) |  | / |  |
| SII |  |  |  |  |  |  |  |
| <314.98 | 79 | 1 | 0.012 | / | 0.064 | / | 0.109 |
| ≥314.98 | 139 | 0.65 (0.47-0.91) |  | / |  | / |  |
| PLR |  |  |  |  |  |  |  |
| <84.57 | 29 | 1 | 0.001 | 1 | 0.003 | 1 | 0.002 |
| ≥84.57 | 189 | 0.49 (0.32-0.75) |  | 0.51 (0.33-0.79) |  | 0.50 (0.32-0.77) |  |
| NLR |  |  |  |  |  |  |  |
| <1.23 | 45 | 1 | 0.016 | 1 | 0.021 | / | 0.086 |
| ≥1.23 | 173 | 0.62 (0.43-0.92) |  | 0.63 (0.43-0.93) |  | / |  |
| CRP |  |  |  |  |  |  |  |
| <3.45 | 151 | 1 | 0.129 | / | 0.154 | / | 0.084 |
| ≥3.45 | 67 | 0.68 (0.41-1.12) |  | / |  | / |  |
| CAR |  |  |  |  |  |  |  |
| <0.082 | 162 | 1 | 0.292 | / | 0.184 | / | 0.084 |
| ≥0.082 | 56 | 0.79 (0.51-1.22) |  | / |  | / |  |
| LCR |  |  |  |  |  |  |  |
| <4477.93 | 42 | 1 | 0.224 | / | 0.220 | / | 0.243 |
| ≥4477.93 | 176 | 1.38 (0.82-2.33) |  | / |  | / |  |
| IBI |  |  |  |  |  |  |  |
| <0.53 | 156 | 1 | 0.244 | / | 0.471 | / | 0.158 |
| ≥0.53 | 62 | 0.78 (0.51-1.19) |  | / |  | / |  |
| AGR |  |  |  |  |  |  |  |
| <1.51 | 44 | 1 | 0.082 | / | 0.438 | / | 0.679 |
| ≥1.51 | 174 | 0.72 (0.49-1.04) |  | / |  | / |  |
| PNI |  |  |  |  |  |  |  |
| <52.09 | 143 | 1 | 0.049 | 1 | 0.028 | 1 | 0.012 |
| ≥52.09 | 75 | 1.41 (1.00-1.98) |  | 1.47 (1.04-2.08) |  | 1.58 (1.11-2.26) |  |
| GLR |  |  |  |  |  |  |  |
| <2.87 | 135 | 1 | 0.038 | 1 | 0.018 | / | 0.081 |
| ≥2.87 | 83 | 0.70 (0.50-0.98) |  | 0.66 (0.47-0.93) |  | / |  |
| GNRI |  |  |  |  |  |  |  |
| <99.32 | 54 | 1 | 0.036 | / | 0.339 | / | 0.201 |
| ≥99.32 | 164 | 0.68 (0.47-0.98) |  | / |  | / |  |
| mGNRI |  |  |  |  |  |  |  |
| <44.57 | 43 | 1 | 0.135 | / | 0.405 | / | 0.281 |
| ≥44.57 | 175 | 0.69 (0.43-1.12) |  | / |  | / |  |
| NRI |  |  |  |  |  |  |  |
| <100.57 | 56 | 1 | 0.033 | / | 0.292 | / | 0.195 |
| ≥100.57 | 162 | 0.67 (0.47-0.97) |  | / |  | / |  |
| Creatinine |  |  |  |  |  |  |  |
| <41.1 | 26 | 1 | 0.029 | 1 | 0.020 | 1 | 0.011 |
| ≥41.1 | 192 | 0.60 (0.37-0.95) |  | 0.57 (0.36-0.92) |  | 0.53 (0.32-0.86) |  |
| Cystatin C |  |  |  |  |  |  |  |
| <0.86 | 76 | 1 | 4.11×10^-4^ | 1 | 0.040 | / | 0.119 |
| ≥0.86 | 142 | 1.94 (1.34-2.81) |  | 1.56 (1.02-2.39) |  | / |  |
| SI |  |  |  |  |  |  |  |
| <83.9 | 191 | 1 | 0.001 | 1 | 0.007 | 1 | 0.020 |
| ≥83.9 | 27 | 0.34 (0.19-0.63) |  | 0.42 (0.22-0.79) |  | 0.46 (0.24-0.88) |  |

Model 0, unadjusted model; Model 1, adjusted by age and BMI (except for ALI); Model 2, adjusted by age, BMI (except for ALI), site of onset, diagnostic delay, and ALSFRS-R score.

95% CI, 95% confidence interval; AGR, albumin‐to‐globulin ratio; ALI, advanced lung cancer inflammation index; ALS, amyotrophic lateral sclerosis; BMI, body mass index; CAR, C‐reactive protein‐to‐albumin ratio; CRP: C‐reactive protein; GLR, glucose‐to‐lymphocyte ratio; GNRI, geriatric nutritional risk index; IBI: inflammatory burden index; LCR, lymphocyte‐to‐C‐reactive protein ratio; mGNRI, modified geriatric nutritional risk index; NLR, neutrophil‐to‐lymphocyte ratio; NRI, nutritional risk index; PLR, platelet‐to‐lymphocyte ratio; PNI, prognostic nutritional index; SI, sarcopenia index; SII, systemic immune‐inflammation index.

**Table S4. Univariate and Multivariate Cox survival analysis in patients with bulbar onset.**

|  |  | Model 0 | | Model 1 | | Model 2 | |
| --- | --- | --- | --- | --- | --- | --- | --- |
| Variables | Number of patients | Hazard Ratio (95% CI) | p-value | Hazard Ratio (95% CI) | p-value | Hazard Ratio (95% CI) | p-value |
| ALI |  |  |  |  |  |  |  |
| <35.50 | 13 | 1 | 0.126 | / | 0.762 | / | 0.630 |
| ≥35.50 | 75 | 0.60 (0.32-1.15) |  | / |  | / |  |
| SII |  |  |  |  |  |  |  |
| <317.85 | 38 | 1 | 0.048 | / | 0.071 | / | 0.322 |
| ≥317.85 | 50 | 0.61 (0.38-0.99) |  | / |  | / |  |
| PLR |  |  |  |  |  |  |  |
| <102.94 | 29 | 1 | 0.033 | 1 | 0.008 | 1 | 0.042 |
| ≥102.94 | 59 | 0.59 (0.36-0.96) |  | 0.50 (0.30-0.84) |  | 0.59 (0.36-0.98) |  |
| NLR |  |  |  |  |  |  |  |
| <2.03 | 64 | 1 | 0.161 | 1 | 0.011 | 1 | 0.035 |
| ≥2.03 | 24 | 0.66 (0.38-1.18) |  | 0.44 (0.23-0.83) |  | 0.50 (0.26-0.95) |  |
| CRP |  |  |  |  |  |  |  |
| <3.16 | 53 | 1 | 0.229 | / | 0.172 | / | 0.075 |
| ≥3.16 | 35 | 0.71 (0.410-1.24) |  | / |  | / |  |
| CAR |  |  |  |  |  |  |  |
| <0.073 | 53 | 1 | 0.250 | / | 0.147 | / | 0.078 |
| ≥0.073 | 35 | 0.72 (0.42-1.26) |  | / |  | / |  |
| LCR |  |  |  |  |  |  |  |
| <4715.17 | 22 | 1 | 0.325 | / | 0.098 | 1 | 0.031 |
| ≥4715.17 | 66 | 1.40 (0.72-2.72) |  | / |  | 2.18 (1.07-4.45) |  |
| IBI |  |  |  |  |  |  |  |
| <0.62 | 67 | 1 | 0.491 | / | 0.875 | / | 0.795 |
| ≥0.62 | 21 | 0.76 (0.34-1.68) |  | / |  | / |  |
| AGR |  |  |  |  |  |  |  |
| <1.49 | 15 | 1 | 0.200 | / | 0.338 | / | 0.937 |
| ≥1.49 | 73 | 0.67 (0.37-1.24) |  | / |  | / |  |
| PNI |  |  |  |  |  |  |  |
| <50.34 | 46 | 1 | 0.100 | / | 0.323 | / | 0.464 |
| ≥50.34 | 42 | 0.67 (0.42-1.08) |  | / |  | / |  |
| GLR |  |  |  |  |  |  |  |
| <2.53 | 34 | 1 | 0.251 | 1 | 0.013 | 1 | 0.022 |
| ≥2.53 | 54 | 0.75 (0.47-1.22) |  | 0.52 (0.31-0.87) |  | 0.55 (0.33-0.92) |  |
| GNRI |  |  |  |  |  |  |  |
| <101.56 | 33 | 1 | 0.004 | / | 0.157 | / | 0.760 |
| ≥101.56 | 55 | 0.49 (0.30-0.80) |  | / |  | / |  |
| mGNRI |  |  |  |  |  |  |  |
| <55.85 | 45 | 1 | 0.308 | / | 0.057 | / | 0.226 |
| ≥55.85 | 43 | 1.32 (0.77-2.26) |  | / |  | / |  |
| NRI |  |  |  |  |  |  |  |
| <102.76 | 33 | 1 | 0.004 | / | 0.157 | / | 0.760 |
| ≥102.76 | 55 | 0.49 (0.30-0.80) |  | / |  | / |  |
| Creatinine |  |  |  |  |  |  |  |
| <69.0 | 60 | 1 | 0.095 | / | 0.542 | / | 0.686 |
| ≥69.0 | 28 | 1.53 (0.93-2.52) |  | / |  | / |  |
| Cystatin C |  |  |  |  |  |  |  |
| <1.01 | 58 | 1 | 0.006 | / | 0.687 | 1 | 0.011 |
| ≥1.01 | 30 | 1.97 (1.21-3.20) |  | / |  | 2.01 (1.17-3.43) |  |
| SI |  |  |  |  |  |  |  |
| <93.7 | 77 | 1 | 0.086 | 1 | 0.007 | 1 | 0.005 |
| ≥93.7 | 11 | 0.48 (0.21-1.11) |  | 0.28 (0.11-0.71) |  | 0.25 (0.10-0.66) |  |

Model 0, unadjusted model; Model 1, adjusted by age, sex, and BMI (except for ALI); Model 2, adjusted by age, sex, BMI (except for ALI), diagnostic delay, and ALSFRS-R score.

95% CI, 95% confidence interval; AGR, albumin‐to‐globulin ratio; ALI, advanced lung cancer inflammation index; BMI, body mass index; CAR, C‐reactive protein‐to‐albumin ratio; CRP: C‐reactive protein; GLR, glucose‐to‐lymphocyte ratio; GNRI, geriatric nutritional risk index; IBI: inflammatory burden index; LCR, lymphocyte‐to‐C‐reactive protein ratio; mGNRI, modified geriatric nutritional risk index; NLR, neutrophil‐to‐lymphocyte ratio; NRI, nutritional risk index; PLR, platelet‐to‐lymphocyte ratio; PNI, prognostic nutritional index; SI, sarcopenia index; SII, systemic immune‐inflammation index.

**Table S5. Univariate and Multivariate Cox survival analysis in patients with limb onset.**

|  |  | Model 0 | | Model 1 | | Model 2 | |
| --- | --- | --- | --- | --- | --- | --- | --- |
| Variables | Number of patients | Hazard Ratio (95% CI) | p-value | Hazard Ratio (95% CI) | p-value | Hazard Ratio (95% CI) | p-value |
| ALI |  |  |  |  |  |  |  |
| <53.23 | 186 | 1 | 0.048 | / | 0.482 | / | 0.404 |
| ≥53.23 | 232 | 0.79 (0.62-1.00) |  | / |  | / |  |
| SII |  |  |  |  |  |  |  |
| <316.90 | 139 | 1 | 0.841 | / | 0.050 | / | 0.081 |
| ≥316.90 | 279 | 0.84  (0.66-1.08) |  | / |  | / |  |
| PLR |  |  |  |  |  |  |  |
| <84.95 | 72 | 1 | 0.118 | 1 | 0.029 | 1 | 0.007 |
| ≥84.95 | 346 | 0.78 (0.58-1.06) |  | 0.71 (0.52-0.97) |  | 0.65 (0.48-0.89) |  |
| NLR |  |  |  |  |  |  |  |
| <1.18 | 58 | 1 | 0.088 | 1 | 0.024 | / | 0.253 |
| ≥1.18 | 360 | 0.76 (0.55-1.04) |  | 0.69 (0.50-0.95) |  | / |  |
| CRP |  |  |  |  |  |  |  |
| <0.74 | 89 | 1 | 0.143 | / | 0.104 | / | 0.623 |
| ≥0.74 | 329 | 1.38 (0.90-2.13) |  | / |  | / |  |
| CAR |  |  |  |  |  |  |  |
| <0.023 | 116 | 1 | 0.380 | / | 0.389 | / | 0.699 |
| ≥0.023 | 302 | 1.17 (0.83-1.65) |  | / |  | / |  |
| LCR |  |  |  |  |  |  |  |
| <4121.04 | 72 | 1 | 0.135 | / | 0.626 | / | 0.221 |
| ≥4121.04 | 346 | 0.73 (0.49-1.10) |  | / |  | / |  |
| IBI |  |  |  |  |  |  |  |
| <0.57 | 294 | 1 | 0.181 | / | 0.592 | / | 0.759 |
| ≥0.57 | 114 | 0.79 (0.56-1.11) |  | / |  | / |  |
| AGR |  |  |  |  |  |  |  |
| <1.51 | 66 | 1 | 0.008 | / | 0.205 | / | 0.148 |
| ≥1.51 | 352 | 0.67 (0.50-0.90) |  | / |  | / |  |
| PNI |  |  |  |  |  |  |  |
| <46.35 | 48 | 1 | 3.79×10^-4^ | 1 | 0.050 | 1 | 0.011 |
| ≥46.35 | 370 | 0.53 (0.38-0.76) |  | 0.69 (0.48-1.00) |  | 0.63 (0.44-0.90) |  |
| GLR |  |  |  |  |  |  |  |
| <1.96 | 89 | 1 | 0.335 | / | 0.825 | / | 0.585 |
| ≥1.96 | 329 | 1.16 (0.86-1.55) |  | / |  | / |  |
| GNRI |  |  |  |  |  |  |  |
| <98.66 | 101 | 1 | 0.009 | / | 0.098 | / | 0.119 |
| ≥98.66 | 317 | 0.70 (0.53-0.91) |  | / |  | / |  |
| mGNRI |  |  |  |  |  |  |  |
| <43.76 | 72 | 1 | 0.024 | / | 0.292 | / | 0.214 |
| ≥43.76 | 346 | 0.63 (0.42-0.94) |  | / |  | / |  |
| NRI |  |  |  |  |  |  |  |
| < 99.76 | 100 | 1 | 0.013 | / | 0.124 | / | 0.149 |
| ≥99.76 | 318 | 0.71 (0.54-0.93) |  | / |  | / |  |
| Creatinine |  |  |  |  |  |  |  |
| <53.2 | 133 | 1 | 0.002 | 1 | 2.8×10^-5^ | 1 | <0.0001 |
| ≥53.2 | 285 | 0.67 (0.52-0.86) |  | 0.56 (0.42-0.73) |  | 0.47 (0.36-0.63) |  |
| Cystatin C |  |  |  |  |  |  |  |
| <1.05 | 256 | 1 | 1.24×10^-4^ | 1 | 0.005 | / | 0.091 |
| ≥1.05 | 162 | 1.60 (1.26-2.03) |  | 1.43 (1.12-1.83) |  | / |  |
| SI |  |  |  |  |  |  |  |
| <59.8 | 114 | 1 | <0.0001 | 1 | <0.0001 | 1 | <0.0001 |
| ≥59.8 | 304 | 0.49 (0.38-0.63) |  | 0.49 (0.37-0.64) |  | 0.47 (0.36-0.62) |  |

Model 0, unadjusted model; Model 1, adjusted by age, sex, and BMI (except for ALI); Model 2, adjusted by age, sex, BMI (except for ALI), diagnostic delay, and ALSFRS-R score.

95% CI, 95% confidence interval; AGR, albumin‐to‐globulin ratio; ALI, advanced lung cancer inflammation index; BMI, body mass index; CAR, C‐reactive protein‐to‐albumin ratio; CRP: C‐reactive protein; GLR, glucose‐to‐lymphocyte ratio; GNRI, geriatric nutritional risk index; IBI: inflammatory burden index; LCR, lymphocyte‐to‐C‐reactive protein ratio; mGNRI, modified geriatric nutritional risk index; NLR, neutrophil‐to‐lymphocyte ratio; NRI, nutritional risk index; PLR, platelet‐to‐lymphocyte ratio; PNI, prognostic nutritional index; SI, sarcopenia index; SII, systemic immune‐inflammation index.

**Table S6. Univariate and Multivariate Cox survival analysis in patients aged<60 years.**

|  |  | Model 0 | | Model 1 | | Model 2 | |
| --- | --- | --- | --- | --- | --- | --- | --- |
| Variables | Number of patients | Hazard Ratio (95% CI) | p-value | Hazard Ratio (95% CI) | p-value | Hazard Ratio (95% CI) | p-value |
| ALI |  |  |  |  |  |  |  |
| <82.14 | 278 | 1 | 0.373 | / | 0.372 | / | 0.252 |
| ≥82.14 | 56 | 1.17 (0.83-1.65) |  | / |  | / |  |
| SII |  |  |  |  |  |  |  |
| <317.85 | 121 | 1 | 0.022 | / | 0.062 | 1 | 0.010 |
| ≥317.85 | 213 | 0.72  (0.54-0.95) |  | / |  | 0.69  (0.52-0.92) |  |
| PLR |  |  |  |  |  |  |  |
| <84.95 | 65 | 1 | 0.014 | 1 | 0.030 | 1 | 0.002 |
| ≥84.95 | 269 | 0.66 (0.48-0.92) |  | 0.69 (0.50-0.97) |  | 0.59 (0.42-0.82) |  |
| NLR |  |  |  |  |  |  |  |
| <1.16 | 49 | 1 | 0.087 | / | 0.216 | / | 0.742 |
| ≥1.16 | 285 | 0.73 (0.51-1.05) |  | / |  | / |  |
| CRP |  |  |  |  |  |  |  |
| <3.44 | 226 | 1 | 0.198 | / | 0.190 | / | 0.080 |
| ≥3.44 | 108 | 0.76 (0.49-1.16) |  | / |  | / |  |
| CAR |  |  |  |  |  |  |  |
| <0.014 | 63 | 1 | 0.155 | / | 0.101 | / | 0.939 |
| ≥0.014 | 271 | 1.56 (0.85-2.88) |  | / |  | / |  |
| LCR |  |  |  |  |  |  |  |
| <12742.22 | 153 | 1 | 0.272 | / | 0.228 | / | 0.064 |
| ≥12742.22 | 181 | 1.20 (0.87-1.64) |  | / |  | / |  |
| IBI |  |  |  |  |  |  |  |
| <0.72 | 267 | 1 | 0.289 | / | 0.297 | / | 0.188 |
| ≥0.72 | 67 | 0.78 (0.50-1.23) |  | / |  | / |  |
| AGR |  |  |  |  |  |  |  |
| <1.51 | 33 | 1 | 0.055 | / | 0.074 | 1 | 0.005 |
| ≥1.51 | 301 | 0.67 (0.45-1.01) |  | / |  | 0.55 (0.36-0.83) |  |
| PNI |  |  |  |  |  |  |  |
| <46.90 | 31 | 1 | 0.012 | 1 | 0.006 | 1 | 0.003 |
| ≥46.90 | 303 | 0.59 (0.38-0.89) |  | 0.55 (0.36-0.84) |  | 0.51 (0.33-0.80) |  |
| GLR |  |  |  |  |  |  |  |
| <3.62 | 295 | 1 | 0.168 | / | 0.201 | / | 0.522 |
| ≥3.62 | 39 | 0.73 (0.46-1.14) |  | / |  | / |  |
| GNRI |  |  |  |  |  |  |  |
| <98.71 | 59 | 1 | 0.005 | 1 | 0.045 | 1 | 0.021 |
| ≥98.71 | 275 | 0.61 (0.43-0.86) |  | 0.69 (0.48-0.99) |  | 0.66 (0.47-0.94) |  |
| mGNRI |  |  |  |  |  |  |  |
| <44.57 | 67 | 1 | 0.171 | / | 0.732 | / | 0.479 |
| ≥44.57 | 267 | 0.75 (0.49-1.14) |  | / |  | / |  |
| NRI |  |  |  |  |  |  |  |
| < 99.73 | 55 | 1 | 0.027 | / | 0.171 | / | 0.221 |
| ≥99.73 | 279 | 0.67 (0.46-0.95) |  | / |  | / |  |
| Creatinine |  |  |  |  |  |  |  |
| <51.4 | 88 | 1 | 0.002 | 1 | 2.92×10^-4^ | 1 | 3.8×10^-5^ |
| ≥51.4 | 246 | 0.63 (0.46-0.84) |  | 0.54 (0.39-0.75) |  | 0.50 (0.36-0.69) |  |
| Cystatin C |  |  |  |  |  |  |  |
| <0.86 | 104 | 1 | 0.016 | 1 | 0.004 | 1 | 0.010 |
| ≥0.86 | 230 | 1.47 (1.08-2.01) |  | 1.59 (1.16-2.18) |  | 1.53 (1.11-2.11) |  |
| SI |  |  |  |  |  |  |  |
| <54.9 | 44 | 1 | 2.3×10^-5^ | 1 | 4.0×10^-6^ | 1 | <0.0001 |
| ≥54.9 | 290 | 0.48 (0.34-0.67) |  | 0.44 (0.31-0.62) |  | 0.32 (0.22-0.47) |  |

Model 0, unadjusted model; Model 1, adjusted by sex, and BMI (except for ALI); Model 2, adjusted by sex, BMI (except for ALI), site of onset, diagnostic delay, and ALSFRS-R score.

95% CI, 95% confidence interval; AGR, albumin‐to‐globulin ratio; ALI, advanced lung cancer inflammation index; BMI, body mass index; CAR, C‐reactive protein‐to‐albumin ratio; CRP: C‐reactive protein; GLR, glucose‐to‐lymphocyte ratio; GNRI, geriatric nutritional risk index; IBI: inflammatory burden index; LCR, lymphocyte‐to‐C‐reactive protein ratio; mGNRI, modified geriatric nutritional risk index; NLR, neutrophil‐to‐lymphocyte ratio; NRI, nutritional risk index; PLR, platelet‐to‐lymphocyte ratio; PNI, prognostic nutritional index; SI, sarcopenia index; SII, systemic immune‐inflammation index.

**Table S7. Univariate and Multivariate Cox survival analysis in patients aged≥60 years.**

|  |  | Model 0 | | Model 1 | | Model 2 | |
| --- | --- | --- | --- | --- | --- | --- | --- |
| Variables | Number of patients | Hazard Ratio (95% CI) | p-value | Hazard Ratio (95% CI) | p-value | Hazard Ratio (95% CI) | p-value |
| ALI |  |  |  |  |  |  |  |
| <75.08 | 142 | 1 | 0.198 | / | 0.197 | / | 0.195 |
| ≥75.08 | 30 | 1.33 (0.86-2.03) |  | / |  | / |  |
| SII |  |  |  |  |  |  |  |
| <767.26 | 152 | 1 | 0.185 | / | 0.182 | / | 0.343 |
| ≥767.26 | 20 | 0.69  (0.40-1.20) |  | / |  | / |  |
| PLR |  |  |  |  |  |  |  |
| <149.10 | 118 | 1 | 0.316 | / | 0.316 | / | 0.301 |
| ≥149.10 | 54 | 1.20 (0.84-1.72) |  | / |  | / |  |
| NLR |  |  |  |  |  |  |  |
| <1.50 | 47 | 1 | 0.102 | / | 0.100 | / | 0.260 |
| ≥1.50 | 125 | 0.73 (0.51-1.06) |  | / |  | / |  |
| CRP |  |  |  |  |  |  |  |
| <0.74 | 36 | 1 | 0.116 | / | 0.113 | / | 0.222 |
| ≥0.74 | 136 | 1.62 (0.89-2.95) |  | / |  | / |  |
| CAR |  |  |  |  |  |  |  |
| <0.083 | 124 | 1 | 0.159 | / | 0.158 | / | 0.344 |
| ≥0.083 | 48 | 0.74 (0.48-1.13) |  | / |  | / |  |
| LCR |  |  |  |  |  |  |  |
| <16624.40 | 109 | 1 | 0.203 | / | 0.202 | / | 0.253 |
| ≥16624.40 | 63 | 0.77 (0.52-1.15) |  | / |  | / |  |
| IBI |  |  |  |  |  |  |  |
| <0.32 | 89 | 1 | 0.405 | / | 0.405 | / | 0.334 |
| ≥0.32 | 83 | 0.86 (0.59-1.23) |  | / |  | / |  |
| AGR |  |  |  |  |  |  |  |
| <1.99 | 151 | 1 | 0.140 | / | 0.138 | / | 0.898 |
| ≥1.99 | 21 | 1.46 (0.88-2.40) |  | / |  | / |  |
| PNI |  |  |  |  |  |  |  |
| <54.00 | 149 | 1 | 0.002 | 1 | 0.002 | 1 | 1.06×10^-4^ |
| ≥54.00 | 23 | 2.11 (1.30-3.41) |  | 2.11 (1.30-3.41) |  | 2.76 (1.65-4.60) |  |
| GLR |  |  |  |  |  |  |  |
| <1.83 | 16 | 1 | 0.013 | 1 | 0.013 | 1 | 0.006 |
| ≥1.83 | 156 | 0.50 (0.29-0.86) |  | 0.50 (0.29-0.86) |  | 0.46 (0.26-0.80) |  |
| GNRI |  |  |  |  |  |  |  |
| <97.98 | 49 | 1 | 0.190 | / | 0.189 | / | 0.160 |
| ≥97.98 | 123 | 1.29 (0.88-1.89) |  | / |  | / |  |
| mGNRI |  |  |  |  |  |  |  |
| <61.55 | 138 | 1 | 0.104 | / | 0.100 | / | 0.131 |
| ≥61.55 | 34 | 0.60 (0.32-1.11) |  | / |  | / |  |
| NRI |  |  |  |  |  |  |  |
| < 95.62 | 16 | 1 | 0.213 | / | 0.210 | / | 0.605 |
| ≥95.62 | 156 | 0.69 (0.39-1.23) |  | / |  | / |  |
| Creatinine |  |  |  |  |  |  |  |
| <75.7 | 153 | 1 | 0.132 | / | 0.129 | / | 0.401 |
| ≥75.7 | 19 | 1.48 (0.89-2.47) |  | / |  | / |  |
| Cystatin C |  |  |  |  |  |  |  |
| <1.04 | 70 | 1 | 0.039 | 1 | 0.039 | / | 0.091 |
| ≥1.04 | 102 | 1.45 (1.02-2.06) |  | 1.45 (1.02-2.06) |  | / |  |
| SI |  |  |  |  |  |  |  |
| <56.9 | 42 | 1 | 0.123 | / | 0.121 | 1 | 0.020 |
| ≥56.9 | 130 | 0.74 (0.51-1.08) |  | / |  | 0.62 (0.41-0.93) |  |

Model 0, unadjusted model; Model 1, adjusted by sex, and BMI (except for ALI); Model 2, adjusted by sex, BMI (except for ALI), site of onset, diagnostic delay, and ALSFRS-R score.

95% CI, 95% confidence interval; AGR, albumin‐to‐globulin ratio; ALI, advanced lung cancer inflammation index; BMI, body mass index; CAR, C‐reactive protein‐to‐albumin ratio; CRP: C‐reactive protein; GLR, glucose‐to‐lymphocyte ratio; GNRI, geriatric nutritional risk index; IBI: inflammatory burden index; LCR, lymphocyte‐to‐C‐reactive protein ratio; mGNRI, modified geriatric nutritional risk index; NLR, neutrophil‐to‐lymphocyte ratio; NRI, nutritional risk index; PLR, platelet‐to‐lymphocyte ratio; PNI, prognostic nutritional index; SI, sarcopenia index; SII, systemic immune‐inflammation index.

**Table S8. The C-index of 17 indicators for ALS patients stratified by sex.**

| Indicators | Men | | | Women | |
| --- | --- | --- | --- | --- | --- |
|  | C-index (95% CI) | p-value | | C-index (95% CI) | p-value |
| ALI | 0.56 (0.51-0.60) | | 0.266 | 0.51 (0.46-0.56) | 0.005 |
| SII | 0.52 (0.47-0.56) | | 0.019 | 0.54 (0.49-0.59 ) | 0.036 |
| PLR | 0.53 (0.48-0.57) | | 0.054 | 0.55 (0.50-0.60 ) | 0.076 |
| NLR | 0.54 (0.49-0.58) | | 0.092 | 0.53 (0.48-0.59) | 0.024 |
| CRP | 0.50 (0.45-0.55) | | 0.019 | 0.53 (0.47-0.58) | 0.011 |
| CAR | 0.51 (0.46-0.57 ) | | 0.038 | 0.52 (0.47-0.58 ) | 0.008 |
| LCR | 0.52 (0.47-0.58) | | 0.077 | 0.54 (0.48-0.59 ) | 0.015 |
| AGR | 0.55 (0.50-0.60 ) | | 0.182 | 0.52 (0.47-0.57 ) | 0.003 |
| IBI | 0.52 (0.47-0.57) | | 0.052 | 0.53 (0.47-0.59) | 0.011 |
| PNI | 0.56 (0.51-0.60 ) | | 0.298 | 0.52 (0.46-0.57) | 0.010 |
| GLR | 0.55 (0.50-0.60) | | 0.226 | 0.55 (0.49-0.60) | 0.056 |
| GNRI | 0.56 (0.51-0.60 ) | | 0.229 | 0.52 (0.47-0.57) | 0.005 |
| mGNRI | 0.51 (0.46-0.56) | | 0.035 | 0.52 (0.46-0.57) | 0.005 |
| NRI | 0.56 (0.51-0.60 ) | | 0.229 | 0.52 (0.47-0.57) | 0.005 |
| Creatinine | 0.54 (0.50-0.59) | | 0.103 | 0.55 (0.50-0.60 ) | 0.005 |
| Cystatin C | 0.57 (0.52-0.61) | | 0.336 | 0.60 (0.55-0.65 ) | 0.410 |
| SI | 0.59 (0.55-0.63) | | NA | 0.62  (0.57- 0.67) | NA |

95% CI, 95% confidence interval; AGR, albumin‐to‐globulin ratio; ALI, advanced lung cancer inflammation index; ALS, amyotrophic lateral sclerosis; CAR, C‐reactive protein‐to‐albumin ratio; CRP: C‐reactive protein; GLR, glucose‐to‐lymphocyte ratio; GNRI, geriatric nutritional risk index; IBI: inflammatory burden index; LCR, lymphocyte‐to‐C‐reactive protein ratio; mGNRI, modified geriatric nutritional risk index; NLR, neutrophil‐to‐lymphocyte ratio; NRI, nutritional risk index; PLR, platelet‐to‐lymphocyte ratio; PNI, prognostic nutritional index; SI, sarcopenia index; SII, systemic immune‐inflammation index. The p-value indicates the difference between the other indicators and SI.

**Table S9. The C-index of 17 indicators for ALS patients stratified by site of onset.**

| Indicators | Bulbar onset | | Limb onset | |
| --- | --- | --- | --- | --- |
|  | C-index (95% CI) | p-value | C-index (95% CI) | p-value |
| ALI | 0.54 (0.46-0.62 ) | 0.761 | 0.53 (0.49-0.57 ) | 0.003 |
| SII | 0.50 (0.43-0.58 ) | 0.346 | 0.50 (0.46-0.54) | <0.001 |
| PLR | 0.54 (0.46-0.61) | 0.753 | 0.51 (0.47-0.55) | <0.001 |
| NLR | 0.51 (0.42-0.59) | 0.347 | 0.52 (0.48-0.56 ) | <0.001 |
| CRP | 0.57 (0.49-0.65 ) | 0.779 | 0.51 (0.46-0.55) | <0.001 |
| CAR | 0.54 (0.46-0.63) | 0.669 | 0.51 (0.46-0.55) | <0.001 |
| LCR | 0.54 (0.45-0.62) | 0.661 | 0.50 (0.46-0.54 ) | <0.001 |
| AGR | 0.53 (0.45-0.62) | 0.720 | 0.53 (0.50-0.57 ) | <0.001 |
| IBI | 0.54 (0.46-0.62) | 0.679 | 0.51 (0.47-0.56) | <0.001 |
| PNI | 0.55 (0.47-0.63) | 0.847 | 0.52 (0.48-0.56 ) | <0.001 |
| GLR | 0.52 (0.44-0.60) | 0.533 | 0.51 (0.47-0.55) | <0.001 |
| GNRI | 0.59 (0.52-0.66) | 0.479 | 0.53 (0.49-0.57 ) | <0.001 |
| mGNRI | 0.54 (0.46-0.63 ) | 0.727 | 0.51 (0.47-0.55) | <0.001 |
| NRI | 0.59 (0.52-0.66 ) | 0.479 | 0.53 (0.49-0.57 ) | <0.001 |
| Creatinine | 0.56 (0.49-0.63 ) | 0.959 | 0.55 (0.51-0.58 ) | <0.001 |
| Cystatin C | 0.62 (0.54-0.70) | 0.111 | 0.58 (0.54-0.62 ) | 0.177 |
| SI | 0.56 (0.48-0.63 ) | NA | 0.61 (0.58-0.65 ) | NA |

95% CI, 95% confidence interval; AGR, albumin‐to‐globulin ratio; ALI, advanced lung cancer inflammation index; ALS, amyotrophic lateral sclerosis; CAR, C‐reactive protein‐to‐albumin ratio; CRP: C‐reactive protein; GLR, glucose‐to‐lymphocyte ratio; GNRI, geriatric nutritional risk index; IBI: inflammatory burden index; LCR, lymphocyte‐to‐C‐reactive protein ratio; mGNRI, modified geriatric nutritional risk index; NLR, neutrophil‐to‐lymphocyte ratio; NRI, nutritional risk index; PLR, platelet‐to‐lymphocyte ratio; PNI, prognostic nutritional index; SI, sarcopenia index; SII, systemic immune‐inflammation index. The p-value indicates the difference between the other indicators and SI.

**Table S10. The C-index of 17 indicators for ALS patients stratified by age.**

| Indicators | Age<60 | | Age≥60 | |
| --- | --- | --- | --- | --- |
|  | C-index (95% CI) | p-value | C-index (95% CI) | p-value |
| ALI | 0.53 (0.49-0.58 ) | 0.047 | 0.53 (0.47-0.58) | 0.667 |
| SII | 0.52 (0.48-0.57) | 0.032 | 0.52 (0.46-0.58) | 0.792 |
| PLR | 0.54 (0.50-0.59) | 0.124 | 0.50 (0.45-0.56 ) | 0.868 |
| NLR | 0.51 (0.47-0.56 ) | 0.006 | 0.53 (0.47-0.59 ) | 0.623 |
| CRP | 0.53 (0.48-0.58) | 0.045 | 0.51 (0.46-0.57) | 0.860 |
| CAR | 0.51 (0.46-0.57 ) | 0.019 | 0.52 (0.46-0.58) | 0.765 |
| LCR | 0.52 (0.47-0.57 ) | 0.027 | 0.51 (0.45-0.57) | 0.959 |
| AGR | 0.51 (0.47-0.56 ) | 0.006 | 0.51 (0.45-0.56 ) | 0.922 |
| IBI | 0.51 (0.46-0.56) | 0.015 | 0.52 (0.46-0.58) | 0.803 |
| PNI | 0.52 (0.47-0.56 ) | 0.008 | 0.52 (0.46-0.58 ) | 0.801 |
| GLR | 0.51 (0.46-0.55 ) | 0.004 | 0.50 (0.44-0.56) | 0.853 |
| GNRI | 0.54 (0.50-0.58) | 0.074 | 0.52 (0.47-0.57 ) | 0.852 |
| mGNRI | 0.52 (0.47-0.57) | 0.022 | 0.51 (0.45-0.56 ) | 0.964 |
| NRI | 0.54 (0.50-0.58 ) | 0.072 | 0.52 (0.47-0.57) | 0.858 |
| Creatinine | 0.55 (0.50-0.59 ) | 0.012 | 0.51 (0.45-0.56) | 0.954 |
| Cystatin C | 0.56 (0.51-0.60 ) | 0.198 | 0.55 (0.50-0.61 ) | 0.225 |
| SI | 0.59 (0.55-0.63 ) | NA | 0.51 (0.45-0.57 ) | NA |

95% CI, 95% confidence interval; AGR, albumin‐to‐globulin ratio; ALI, advanced lung cancer inflammation index; ALS, amyotrophic lateral sclerosis; CAR, C‐reactive protein‐to‐albumin ratio; CRP: C‐reactive protein; GLR, glucose‐to‐lymphocyte ratio; GNRI, geriatric nutritional risk index; IBI: inflammatory burden index; LCR, lymphocyte‐to‐C‐reactive protein ratio; mGNRI, modified geriatric nutritional risk index; NLR, neutrophil‐to‐lymphocyte ratio; NRI, nutritional risk index; PLR, platelet‐to‐lymphocyte ratio; PNI, prognostic nutritional index; SI, sarcopenia index; SII, systemic immune‐inflammation index. The p-value indicates the difference between the other indicators and SI.

| Characteristic | Validation cohorts A | Validation cohorts B | p-value |
| --- | --- | --- | --- |
| Number | 304 | 202 | / |
| Sex (Men/Women) | 169/135 | 119/83 | 0.460 |
| Site of onset (Bulbar/Limb) | 50/254 | 38/164 | 0.492 |
| Age (years) | 54.2±10.4 | 54.3±10.6 | 0.856 |
| Diagnostic delay (months) | 11.0 (6.0-18.0) | 11.0 (6.0-18.0) | 0.740 |
| BMI | 23.5±3.1 | 23.3±3.1 | 0.480 |
| ALSFRS-R score | 40 (36-43) | 41 (36-44) | 0.453 |
| Total protein (g/L) | 65.1 (62.3-68.0) | 64.4 (61.4-67.9) | 0.260 |
| Albumin (g/L) | 41.5 (39.4-43.3) | 41.0 (39.3-42.9) | 0.409 |
| Glucose (mmol/L) | 4.70 (4.35-5.07) | 4.72 (4.39-5.17) | 0.383 |
| Neutrophil (×10^9^/L) | 3.25 (2.62-4.02) | 3.33 (2.70-4.12) | 0.278 |
| Lymphocyte (×10^9^/L) | 1.84 (1.50-2.22) | 1.91 (1.57-2.34) | 0.118 |
| Platelets (×10^9^/L) | 216 (186-255) | 216 (178-260) | 0.376 |
| C-reactive protein (mg/L) | 1.10 (1.00-3.27) | 1.10 (1.00-3.19) | 0.274 |
| Creatinine (umol/L) | 60.4 (51.4-69.6) | 60.1 (52.1-69.1) | 0.622 |
| Cystatin C (mg/L) | 0.98 (0.86-1.10) | 0.97 (0.85-1.08) | 0.429 |

**Table S11.** Demographics characteristics of ALS patients between validation cohorts A and validation cohorts B.

BMI, body mass index.

Table S12. Demographic characteristics of the finally included ALS patients and the initially screened ALS patients

| Characteristic | Finally included patients | Initially screened patients | p-value |
| --- | --- | --- | --- |
| Number | 506 | 789 | / |
| Sex (Men/Women) | 288/218 | 461/328 | 0.591 |
| Site of onset (Bulbar/Limb) | 88/418 | 142/647 | 0.781 |
| Age (years) | 54.2±10.5 | 54.0±10.4 | 0.722 |
| Diagnostic delay (months) | 11.0 (6.0-18.0) | 11.0 (6.0-19.0) | 0.543 |
| BMI | 23.4±3.1 | 23.4±3.4 | 0.888 |
| ALSFRS-R score | 40 (36-44) | 40 (36-43) | 0.565 |
| Riluzole (Yes/No) | 230/276 | 370/419 | 0.612 |
| Edaravone (Yes/No) | 344/162 | 504/285 | 0.129 |

**Fig S1.** The Kaplan-Meier curves of the LCR (a), AGR (b), Creatinine (c) and SI (d) in male patients with amyotrophic lateral sclerosis. LCR, lymphocyte‐to‐C‐reactive protein ratio; AGR, albumin‐to‐globulin ratio; SI, sarcopenia index.

**Fig S2.** The Kaplan-Meier curves of the PLR (a), PNI (b), Creatinine (c) and SI (d) in female patients with amyotrophic lateral sclerosis. PLR, platelet‐to‐lymphocyte ratio; PNI, prognostic nutritional index; SI, sarcopenia index.

**Fig S3.** The Kaplan-Meier curves of the PLR (a), NLR (b), LCR (c), GLR (d), Cystatin C (e) and SI (f) in patients with bulbar onset. PLR, platelet‐to‐lymphocyte ratio; NLR, neutrophil‐to‐lymphocyte ratio; LCR, lymphocyte‐to‐C‐reactive protein ratio; GLR, glucose‐to‐lymphocyte ratio; SI, sarcopenia index.

**Fig S4.** The Kaplan-Meier curves of the PLR (a), PNI (b), Creatinine (c) and SI (d) in patients with limb onset. PLR, platelet‐to‐lymphocyte ratio; PNI, prognostic nutritional index; SI, sarcopenia index.

**Fig S5.** The Kaplan-Meier curves of the SII (a), PLR (b), AGR (c), PNI (d), GNRI (e), Creatinine (f), Cystatin C (g) and SI (h) in patients aged<60 years. SII, systemic immune‐inflammation index; PLR, platelet‐to‐lymphocyte ratio; AGR, albumin‐to‐globulin ratio; PNI, prognostic nutritional index; GNRI, geriatric nutritional risk index; SI, sarcopenia index.

**Fig S6.** The Kaplan-Meier curves of the PNI (a), GLR (b), and SI (c) in patients aged**≥**60 years. PLR, platelet‐to‐lymphocyte ratio; GLR, glucose‐to‐lymphocyte ratio; SI, sarcopenia index.

**Fig. S7** The time‐dependent ROC in patients with amyotrophic lateral sclerosis of SI. ROC: receiver operating characteristic; AUC: area under curve; SI, sarcopenia index.
